# Study of specific nanoenvironments containing α-helices in all-α and (α+β)+(α/β) proteins

**DOI:** 10.1371/journal.pone.0200018

**Published:** 2018-07-10

**Authors:** Ivan Mazoni, Luiz César Borro, José Gilberto Jardine, Inácio Henrique Yano, José Augusto Salim, Goran Neshich

**Affiliations:** 1 Embrapa Agricultural Informatics, Campinas, São Paulo, Brazil; 2 Institute of Biology, University of Campinas, Campinas, São Paulo, Brazil; 3 Embrapa Territorial Management, Campinas, São Paulo, Brazil; 4 Research Center on Biodiversity and Computing, University of São Paulo, São Paulo, São Paulo, Brazil; Russian Academy of Medical Sciences, RUSSIAN FEDERATION

## Abstract

Protein secondary structure elements (PSSEs) such as α-helices, β-strands, and turns are the primary building blocks of the tertiary protein structure. Our primary interest here is to reveal the characteristics of the nanoenvironment formed by both PSSEs and their surrounding amino acid residues (AARs), which might contribute to the general understanding of how proteins fold. The characteristics of such nanoenvironments must be specific to each secondary structure element, and we have set our goal here to gather the fullest possible description of the α-helical nanoenvironment. In general, this postulate (the existence of specific nanoenvironments for specific protein substructures/neighbourhoods/regions with distinct functionality) was already successfully explored and confirmed for some protein regions, such as protein-protein interfaces and enzyme catalytic sites. Consequently, PSSEs were the obvious next choice for additional work for further evidence showing that specific nanoenvironments (having characteristics fully describable by means of structural and physical chemical descriptors) do exist for the corresponding and determined intraprotein regions. The nanoenvironment of α-helices (nEoαH) is defined as any region of the protein where this secondary structure element type is detected. The nEoαH, therefore, includes not only the α-helix amino acid residues but also the residues immediately around the α-helix. The hypothesis that motivated this work is that it might in fact be possible to detect a postulated “signal” or “signature” that distinguishes the specific location of α-helices. This “signal” must be discernible by tracking differences in the values of physical, chemical, physicochemical, structural and geometric descriptors immediately before (or after) the PSSE from those in the region along the α-helices. The search for this specific nanoenvironment “signal” was made possible by aligning previously selected α-helices of equal length. Afterward, we calculated the average value, standard deviation and mean square error at each aligned residue position for each selected descriptor. We applied Student’s t-test, the Kolmogorov-Smirnov test and MANOVA statistical tests to the dataset constructed as described above, and the results confirmed that the hypothesized “signal”/“signature” is both existing/identifiable and capable of distinguishing the presence of an α-helix inside the specific nanoenvironment, contextualized as a specific region within the whole protein. However, such conclusion might rarely be reached if only one descriptor is considered at a time. A more accurate signal with broader coverage is achieved only if one applies multivariate analysis, which means that several descriptors (usually approximately 10 descriptors) should be considered at the same time. To a limited extent (up to a maximum of 15% of cases), such conclusion is also possible with only a single descriptor, and the conclusion is also possible in general for up to 50–80% of cases when no less than 5 nonlinear descriptors are selected and considered. Using all the descriptors considered in this work, provided all assumptions about data characteristics for this analysis are met, multivariate analysis regularly reached a coverage and accuracy above 90%. Understanding how secondary structure elements are formed and maintained within a protein structure could enable a more detailed understanding of how proteins reach their final 3D structure and consequently, their function. Likewise, this knowledge may also improve the tools used to determine how good a structure is by means of comparing the “signal” around a selected PSSE with the one obtained from the best (resolution and quality wise) protein structures available.

## Introduction

Crick [1] explained that proteins are uniquely essential to maintaining life. Although proteins can perform almost any type of role in animals, plants or microorganisms, so far, the most studied protein function is definitely its enzymatic role. A precisely folded 3D structure is necessary for a protein to perform its function, and if a protein has been unfolded, e.g., by heat or some chemical agent, the protein will lose its biological function. Before a protein assumes its final and correct 3D structure, the construction of its PSSEs must have been completed.

Anfinsen [2] experimentally confirmed that the amino acid sequence of a protein provides all the necessary information for the protein to assume its precise and final 3D structure. Although currently it is known that this is only a specific case in such an experiment, Anfinsen stimulated several attempts to predict the 3D structure with the amino acid sequence alone. Several methodologies appeared during the last 4–5 decades, such as homology modelling [3–7], ab initio modelling [8–12], and threading [13–17]. Another possible approach to predicting the final 3D structure of a protein is to understand how the protein forms and maintains the different PSSEs during and after folding. Several methods were developed to predict PSSEs. For example, PSSE predictions have been based on circular dichroism data [18–20], on multi-step learning coupled with a prediction of the solvent accessible surface area, and on backbone torsion angles [21–23], as well as on machine learning techniques [24–26]. The results of the aforementioned techniques are much more accurate now than the early results (from the 1980s) for this particular scientific area. For example, the accuracy of these methods increased from 56% in 1983 [27] to more than 80% in 2015 [28].

The pattern of formed hydrogen bonds between the amino and carboxyl groups along the PSSE, together with their ϕ and ψ dihedral angles inside a particular region of the Ramachandran plot [29], fully defines the PSSE. There are three groups of secondary structure elements: helical structures, β-sheets, and turns. Helical structures are subdivided into helix 2.2_7_, α-helix, helix 3_10_, and π-helix. β-sheet structures may be parallel, anti-parallel and β-bridge. Finally, a turn may be a tight *turn*, multiple *turns*, hairpins and *turns* of type I, II, VIII, I’, II,' VIa1, VIa2, VIb, and IV.

Several algorithms explore the regularity of hydrogen-bond patterns of PSSEs and are frequently employed to identify and distinguish PSSE types. The DSSP algorithm uses the hydrogen-bond pattern recognition and the geometric characteristics extracted from the spatial coordinates of the atoms of the amino acid residues to characterize a secondary structure element [30]. The Stride algorithm uses, in addition to the information used by DSSP, the dihedral angle potentials to characterize a secondary structure element [31]. Cuff [32] showed that the DSSP and Stride definitions agree with each other in 95% of the explored cases. An excellent tool to observe the PSSE definition differences is ^Java^Protein Dossier (^J^PD) [33] of the BlueStar STING suite of programs.

Proteins can be grouped into SSE classes using the SCOP structure classification [34]: all-α, all-β, α+β and α/β. “All-α” proteins are those that have only helical secondary structure elements present in the protein (along with some turns and irregular portions) but crucially, no β-sheets. The “all-β” structures are those that have only β-sheets present in the protein (along with some turns and irregular portions) but no helical structures. In α+β proteins, both α-helices and β-strands are present but are largely segregated. The most frequently detected β-strands in the α+β type of proteins are antiparallel ones [35]. In the α/β type of proteins (when the α-helices and β-strands are alternatingly following each other in the protein structure), the β-strands are mostly organized in a parallel fashion [35]. Finally, there are intrinsically disordered proteins, where neither α-helices nor β-strands are present in the protein's 3D structure [36]. Using the Structural Classification of Proteins (SCOP) database [37] and the Protein Data Bank (PDB) [38], we constructed a DataMart, called General SSEs, which performed the following classifications: 1606 protein chains as all-α – 12 containing a single or exclusive helix and 1594 nonexclusive helical chains (meaning they have more than one helix); 19407 protein chains as (α+β)+(α/β)– 99 “exclusive helix” chains and 19308 nonexclusive helix chains (based on PDB data from August 8, 2016).

This work focuses on α-helical elements and their nanoenvironment in the all-α proteins and the α-helices in (α+β)+(α/β) classes of proteins.

Neshich and coworkers [39] introduced the concept of an intraprotein nanoenvironment, and Moraes and coworkers [40] tested this idea for the first time in a protein interface study.

**Fig 1:**
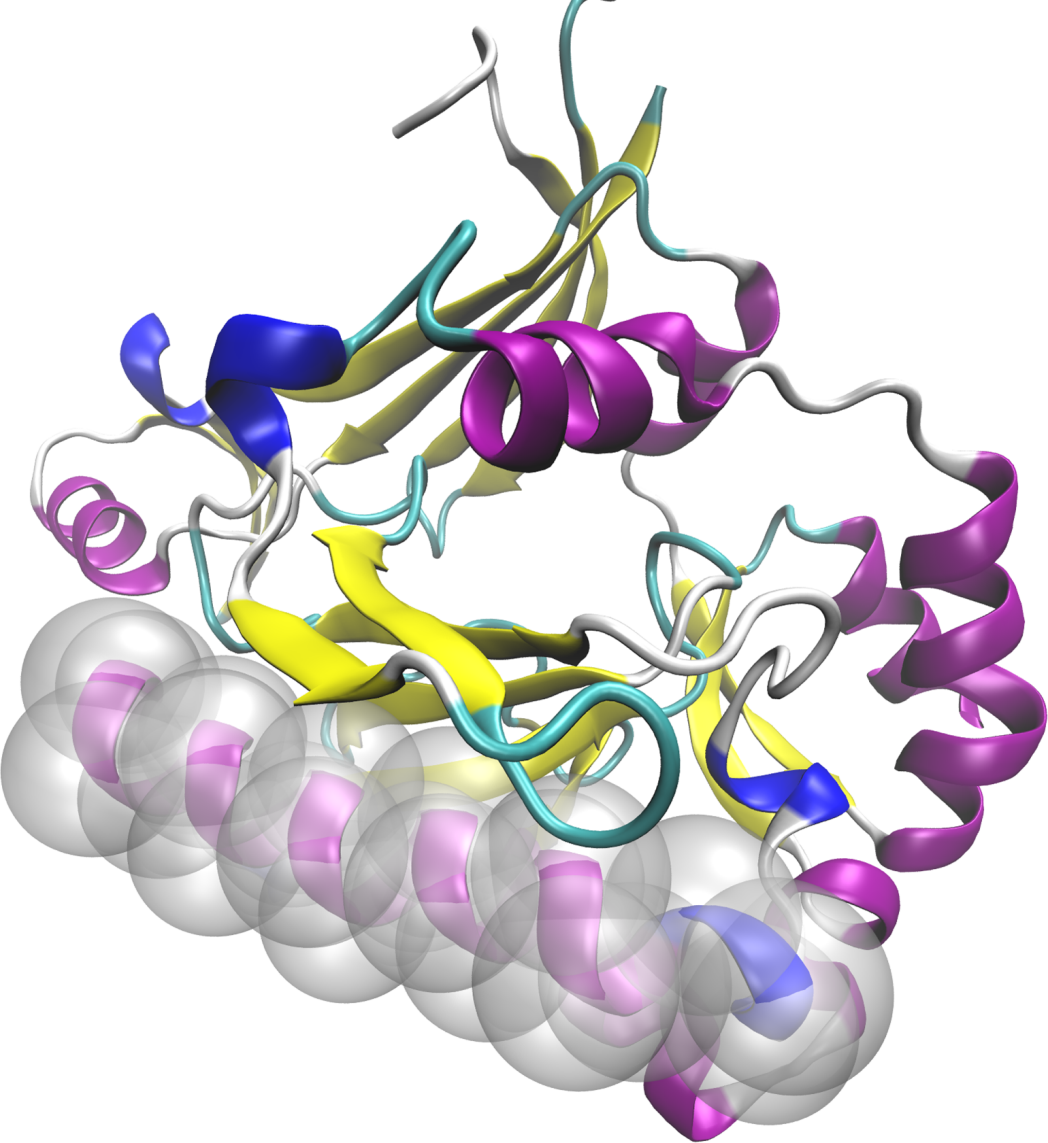
An example of an α-helix (in a specific (α+β) protein) and its nanoenvironment: The synthetic gene encoded DcpS bound to the inhibitor DG157493 (3bl9.pdb) has fourteen α-helices, and each helix has its own nanoenvironment. Highlighted inside the transparent spheres is an α-helix (ribbon, purple). The nanoenvironment includes the amino acid residues of the α-helix and the amino acid residues around the helix that are within reach of the probing sphere, whose radius was previously selected. The pre- and postregions (extension by 32 AARs each) are not shown here for the sake of clarity of the basic definition.

In this work, we considered the set of amino acid residues located within and around α-helices–the α-helical nanoenvironment. The amino acid residues that form the secondary structure element plus the amino acid residues in their vicinity yield a complete neighbourhood with its own, very specific nanoenvironment characteristics (Fig 1).

We used a somewhat arbitrary number, which is still empirically considered the most suitable number, of thirty-two amino acid residues, before and after the secondary structure element in the primary sequence to define the total length of the protein sequence to be studied. The region defined this way is used for analysis so that the corresponding structure fragments can be aligned during a procedure and so that descriptor values can then be inspected for the appearance of the hypothesized specific nanoenvironment “signal”. The spatial inclusion of AARs other than those belonging to the PSSE itself was achieved by defining the radius of a sphere of an “AAR enclosure” drawn from the α-carbon atom of any residue of the selected PSSE. As previously hinted, this procedure was performed for the stretch of thirty-two AARs before a PSSE's N-terminal and after its C-terminal.

Regarding the importance of this work, we certainly argue for possible positive implications of the act of acquiring detailed knowledge about PSSEs and their nanoenvironments and therefore making it possible to perform the following:

- better understand the protein folding process;
- improve existing and design new computational tools for quality validation of the secondary structure element location, extension, and internal geometry in protein structure models obtained either by employing protein structure modelling software, such as Modeller [41] and Swiss-Model [42], or by X-ray crystallography, NMR, and electronic microscopy–and in all cases by inspecting the PSSE environment's characteristics and their “signal-like” behaviour compared to that of a “signal” obtained from the best quality reference structures.

The knowledge about the relationship between protein amino acid sequences, their 3D structure, and their function will enable improvements in many applications, eventually allowing the proposal of substances such as new vaccines, drugs, veterinary drugs, insecticides, all with more efficacy [43]. For example, if one starts from a known genome (human, animal, plant or microorganism) for which it is already possible to obtain an annotated protein sequence, then with the acquired knowledge we are compiling and reporting in this work, the improved secondary structure element prediction will also improve the prediction of the whole protein's 3D structure. Accordingly, this information will lead to finding out how to better develop new protein function inhibitors (e.g., bactericides, pesticides, insecticides, and vaccines). It could therefore be feasible to more precisely simulate enzymatic reactions, protein-protein interactions, and protein-substrate interactions, with all of the simulations being faster, more accurate and quite possibly, less expensive [44].

## Materials and methods

We extracted the data for analysis of the PSSE nanoenvironment from STING_RDB [45]. The STING RDB had 9,320,604,319 records in 98 tables (based on PDB data from August 8, 2016) and included physical, chemical, physicochemical, structural and geometric descriptors (reported in “per amino acid residue” fashion, for each protein chain) of each protein structure in the PDB.

All of the raw data, ready for type of processing that we did in this paper, are available at: https://figshare.com/projects/Structural_and_physical-chemical_characterization_of_alpha-helices/35462

### Two new STING modules for evaluating the PSSE nanoenvironment: PS^3^A and PS^3^DV

This work expanded the Blue Star STING platform [46] by adding two new modules. The first one is called Protein Secondary Structure STING Analyzer (PS^3^A). The PS^3^A, written in the JS and PHP programming languages, allows a user to set some options for the fine tuning of PSSE analysis: PSSE length, consensus type (relative to the definition of the PSSE), redundancy level, and a selector for depicting one of the 69 different descriptors available for the PSSE nanoenvironment. Additionally, the user may obtain the data reliability plot, the sequence “logo” (indicating the local conservation of amino acid residues) and the empirical cumulative distribution function (ECDF) curve. The ECDF curve shows how the descriptor value levels inside the PSSE environment are different from the values outside this environment. Users may access the PS^3^A at https://www.ps3a.cbi.cnptia.embrapa.br/.

PS^3^DataVizualizer is the second new STING module that was added as a visualization tool for PS^3^A, and it offers users the possibility to visualize any of the 69 different STING descriptors available in separate plots that are produced for the nanoenvironments of α-helices (later, we will also have PS^3^DV for β-strands and turns as well). The capabilities of PS^3^DataVizualizer make it possible to observe the PSSE in two ways: exclusive α-helices (only a single helical structure segment is present in the protein's whole structure) and nonexclusive α-helices (more than one helix is present per protein analysed). Users may select any image and keep viewing a carousel of more than two thousand images representing combinations of the selected size of the PSSE and a multitude of protein descriptors drawn from STING_DB. These plots were produced using the R package with a high-quality image generator enabled. Images may be saved by the user for later analysis. We developed PS^3^DV in HTML5 integrated with JS and also used the jQuery and Bootstrap technologies. PS^3^DV is accessible at https://www.ps3dv.cbi.cnptia.embrapa.br/

### Initial hypothesis verification

The hypothesis explored and evaluated here (and contextualized in the biological sense) was as follows: the nanoenvironment descriptor values inside the PSSE are, or are not, statistically equal to the values outside the PSSE. The same hypothesis contextualized statistically was as follows: *H*_0_–the descriptor value distributions are the same, and *H*_1_–the descriptor value distributions are not the same (again: inside vs outside the PSSE nanoenvironment).

Three statistical tests were applied to verify the hypothesis. The first one, the Kolmogorov-Smirnov test (KSt) [47], was used to compare a sample of interest to one with a reference probability distribution. As a nonparametric test of the equality of continuous, the KSt may also be used to compare two samples. The Kolmogorov-Smirnov statistics quantify the distance between the empirical distribution function of the cumulative distribution for the reference distribution and that of the analysed sample or the distance between the two empirical distribution functions of two samples. The empirical distribution function *F*_*n*_ for *n* observations *X*_*i*_ is defined as shown in the equation:

$$F_{n}=\frac{1}{n}\sum_{i=1}^{n}I_{\left[-\infty ,x\right]}(X_{i})$$

where *I*_[−∞,*x*]_(*X*_*i*_) is the indicator function, is equal to 1 if *X*_*i*_ < *x* and is otherwise equal to 0.

The Kolmogorov-Smirnov statistic for *F*_*n*_ is calculated by finding the supremum (or the greatest lower bound) absolute value of the differences between the two samples:

$$D_{n}={sup}_{x}|F_{n}(x)-F(x)|$$

where *sup*_*x*_ is the supremum of the set of distances.

The distance *D*_*n*_ is used to calculate the p-value that indicates whether the numbers differ significantly. The null hypothesis *H*_0_ is rejected if the p-value is close to zero.

The second statistical test applied was the Q-Q plot [48], which we used here to support Student’s t-test [49]. The Q-Q plot is a graphical method for comparing two probability distributions by plotting their quantiles against each other. If the data have a normal distribution, then Student’s t-test may be used. Student’s t-test for different sample sizes may be calculated using the equation below:

$$t=\frac{\bar{x_{1}}-\bar{x_{2}}}{S_{x_{1}x_{2}}\sqrt{\frac{1}{n_{1}}+\frac{1}{n_{2}}}}$$

where $\bar{x_{i}}$ is the sample average, *n*_*i*_ is the sample size, $S_{x_{1}x_{2}}=\sqrt{\frac{(n_{1}-1)S_{x_{1}}^{2}+(n_{2}-1)S_{x_{2}}^{2}}{n_{1}+n_{2}-2}}$, and the degrees of freedom used in this test are *n*_1_ + *n*_2_ – 2.

### Descriptor value sliding window

**Fig 2:**
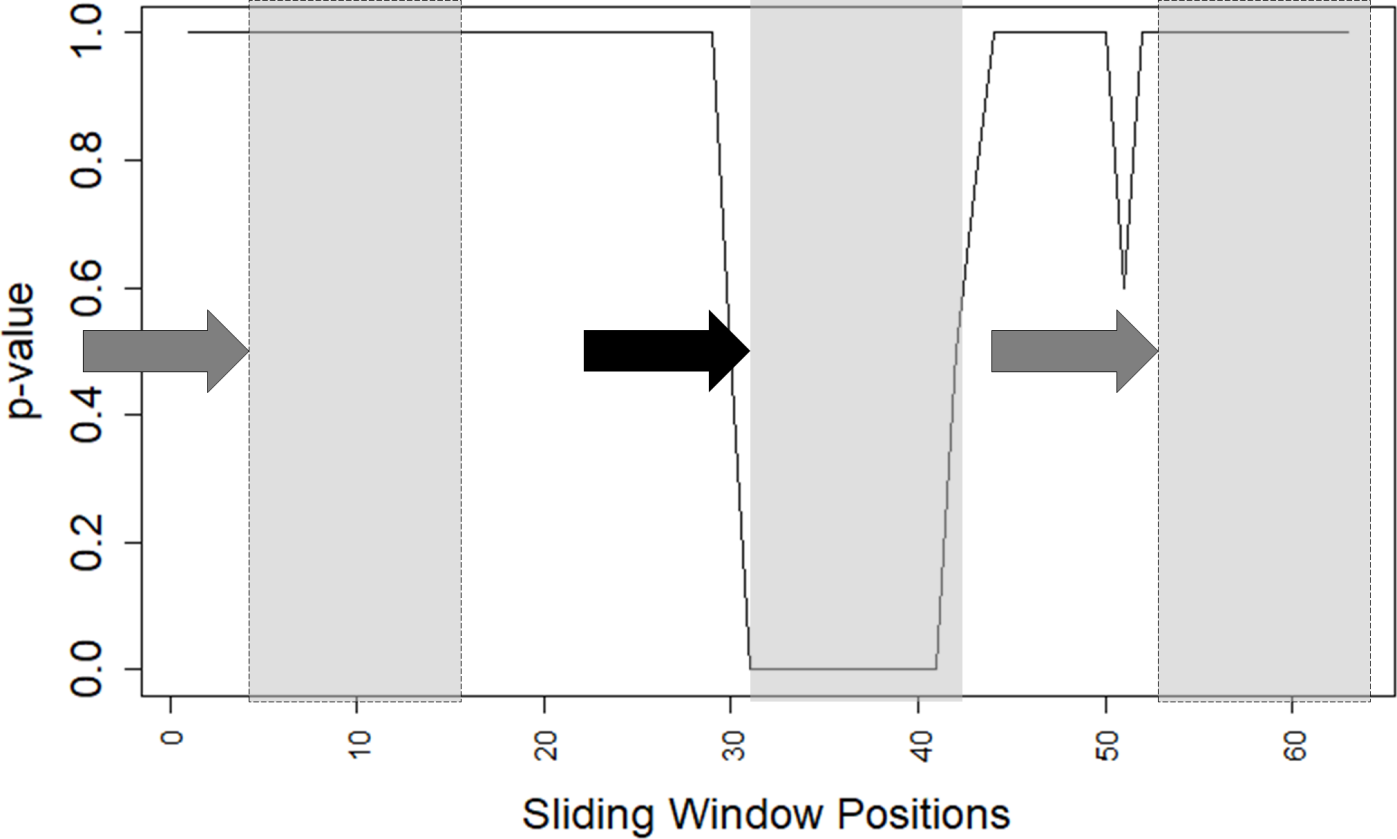
The p-value of Student's t-test evaluation for a selected descriptor value along the “sliding window” for positionally aligned PSSE sequences. The coverage of the sequence containing a PSSE is from the N- to the C-terminal ends (± 32 AAR). The sequences includes the PSSE plus 32 residues before its N-terminal and 32 residues after its C-terminal. The “sliding window” size in this particular case is the same size as the selected PSSE length (12 AAR). Student’s t-test is used for each position of the sliding window. This test measures how much the data inside the “sliding window” differ from the data outside the windows. The p-values are shown along the y-axes. A p-value that approaches zero in any particular region means that within this region, the descriptor values differ from the values outside the region in a statistically significant manner. The arrows indicate the direction of movement for the “sliding window box” (shown here before, at and after the PSSE), and the solid arrow indicates the exact position of the N-terminal of the PSSE. Shadowed boxes indicate the size of the sliding window placed at three specific positions. The region with a p-value approximating zero coincides with the positional alignment of the α-helix that has the exact same size. The sharp invagination around AAR position 52 is not as representative (too short compared to the PSSE under investigation) as the one directly on top and over the whole analysed PSSE.

Another approach used in this work to confirm the existence of the hypothesized “signal” (where the word “signal” is used here to designate “something that shows that something else exists or is likely to happen”) was to run a “sliding window” along the positional/structural alignment for average descriptor values of amino acid residues belonging to the pre-, post- and PSSE regions. For each descriptor, a limited (PSSE size wise) sliding window test was performed by varying the PSSE length between 1 and the maximum number of amino acid residues found (this number can be some of the PSSE sizes found in the STING RDB and actually found in some specific derived DataMarts, which are described below). For such selected lengths, we collected all amino acid residue descriptor values, and the average value was calculated. We then grouped and stored these data using an R script. Student’s t-test was applied while dividing the data into two sets: inside and outside the “sliding window”. If the p-value within the region where the window matches (exactly covers) the PSSE length, approached zero, then the environment/sample inside the window region was considered statistically different from the region located outside that window. The results obtained confirm that the descriptor values inside the PSSE are statistically distinct from the values outside (Fig 2).

### Elimination of redundancies

The presence of redundant sequences inevitably introduces bias in the statistical tests, masking the real variance in the descriptor values along the PSSE positional alignment. We eliminated sequence redundancy (along the PSSE length) at the 95%, 70%, and 50% levels of similarity.

### Building PS^3^A datamarts

**Fig 3:**
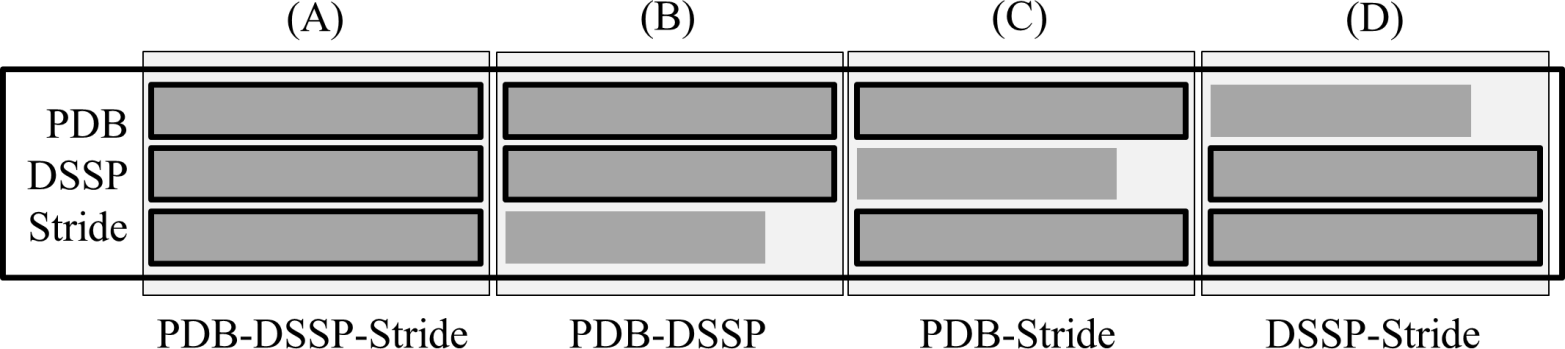
Grouping of same-length α-helices using consensus definitions based on the PDB, DSSP and Stride classifications. There are four possible consensus groups. (A) PDB-DSSP-Stride: when the secondary structure element starts and finishes at the same corresponding amino acid residue location and hence, has the same length according to the PDB, DSSP and Stride definitions. (B), (C) and (D) when the secondary structure elements start but do NOT finish at the same amino acid residue, as defined by one of the three criteria used: PDB-DSSP, PDB-Stride, and DSSP-Stride definitions, respectively.

The first step undertaken in this work was to obtain all the protein structures from STING_RDB containing at least one α-helix. Based on the PDB, DSSP and Stride definitions, the all-α and (α+β)+(α/β) proteins were filtered and stored for further analysis. The second step was to eliminate the protein sequence redundancy at the 95%, 70%, and 50% levels of similarity using CD-HIT software [50]. The third step was to group the α-helices according to the consensus detected among the different algorithms used here to identify/characterize the PSSEs. The most rigid consensus requires a consensus for all three selected algorithms: PDB, DSSP and Stride, i.e., the α-helices should have the same length according to all three definitions. Another possible consensus would be based on the definitions of only two algorithms: PDB-DSSP, PDB-Stride, and DSSP-Stride (Fig 3). The fourth step in this work was to positionally/structurally align the α-helices of equal length. The statistics for such PSSE compilations (tabulated below) and some examples of plots using the PDB-DSSP, PDB-Stride and DSSP-Stride consensus are available in the Supporting Information (Figure A to Figure C in S1 File).

Before proceeding to the next step, the names of the basic datamarts used frequently in this work are listed: DM1, DM1_e, DM1_ne, DM2, DM2_e, and DM2_ne. There is a complete description of these datamarts in Table 1, “Number of Helices of Different Sizes in All α and α+β and α/β Proteins”. “DM1_e” shows the number of helices for each length in all-α proteins for the so-called “exclusive” helices (or just one helix in the whole protein structure). “DM1_ne” shows the same information but for the cases involving more than one helix (nonexclusive helices). DM1 is the sum of DM1_e and DM1_ne. DM2 follows the same nomenclature but considers α+β and α/β proteins.

**Table 1:** Number of helices of different lengths in all α and α+β and α/β proteins.

DM1		DM1_e		DM1_ne		DM2		DM2_e		DM2_ne	
all-α (exc. + nexc.)		all-α (exc.)		all-α (nexc.)		α+β (exc. + nexc.)		α+β (exc.)		α+β (nexc.)	
*Helix size*	*# of helices*	*Helix size*	*# of helices*	*Helix size*	*# of helices*	*Helix size*	*# of helices*	*Helix size*	*# of helices*	*Helix size*	*# of helices*
**5**	81			5	81	5	1463	5	1	5	1462
**6**	177	6	1	6	176	6	1768	6	14	6	1754
**7**	97	7	2	7	95	7	1549	7	10	7	1539
**8**	133	8	1	8	132	8	1173	8	3	8	1170
**9**	62			9	62	9	1212	9	2	9	1210
**10**	100			10	100	10	1619	10	6	10	1613
**11**	107	11	1	11	106	11	1692	11	12	11	1680
**12**	87			12	87	12	1657	12	3	12	1654
**13**	111			13	111	13	1222	13	1	13	1221
**14**	92	14	2	14	90	14	1215	14	5	14	1210
**15**	131	15	1	15	130	15	882	15	6	15	876
**16**	44			16	44	16	643	16	1	16	642
**17**	27			17	27	17	572	17	28	17	544
**18**	50			18	50	18	490	18	1	18	489
**19**	52			19	52	19	392			19	392
**20**	20	20	2	20	18	20	323	20	3	20	320
**21**	21			21	21	21	428	21	1	21	427
**22**	23			22	23	22	194			22	194
**23**	12			23	12	23	99			23	99
**24**	18			24	18	24	123			24	123
**25**	41			25	41	25	126			25	126
**26**	4			26	4	26	61			26	61
**27**	14			27	14	27	106			27	106
**28**	19			28	19	28	56			28	56
**29**	10			29	10	29	84			29	84
**30**	3	30	1	30	2	30	41	30	1	30	40
**31**	9			31	9	31	46			31	46
**32**	18			32	18	32	60			32	60
**33**	12			33	12	33	37			33	37
						34	17			34	17
**35**	4			35	4	35	6			35	6
**36**	2			36	2	36	4			36	4
**37**	1			37	1	37	5			37	5
**38**	1			38	1	38	3			38	3
**39**	1			39	1	39	1			39	1
**40**	2	40	1	40	1	40	4	40	1	40	3
						41	1			41	1
**42**	1			42	1	42	1			42	1
**43**	1			43	1	43	7			43	7
**44**	1			44	1	44	1			44	1
**45**	2			45	2	45	3			45	3
**48**	1			48	1	48	1			48	1
**50**	2			50	2	50	2			50	2
**51**	7			51	7	51	7			51	7
						54	1			54	1
**55**	1			55	1	55	1			55	1
						60	1			60	1
**62**	2			62	2	62	2			62	2
						66	1			66	1
**67**	1			67	1	67	1			67	1
						71	1			71	1
						107	1			107	1
**108**	1			108	1	108	1			108	1
						109	1			109	1
**Total**	**1606**	**Total**	**12**	**Total**	**1594**	**Total**	**19407**	**Total**	**99**	**Total**	**19308**

### Positional/structural alignment of α-helices

**Fig 4:**
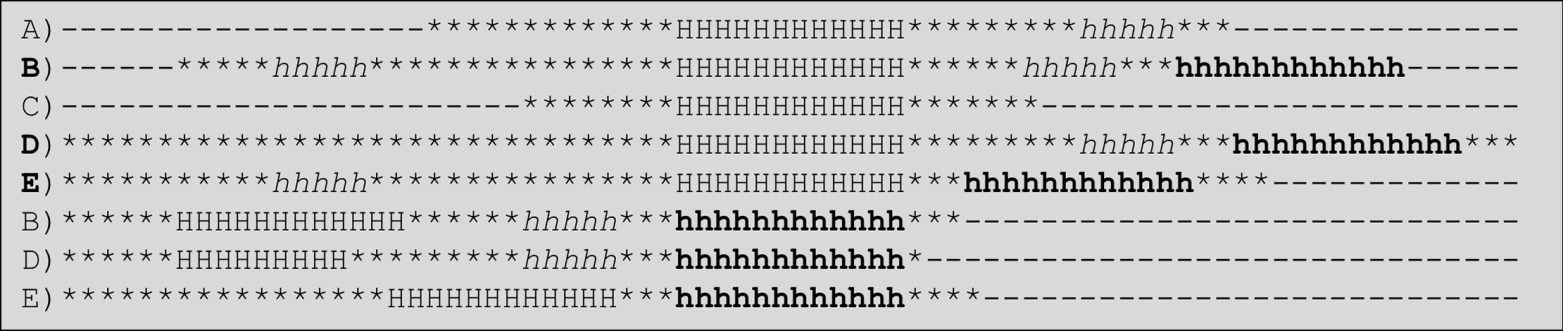
Positional alignment of α-helices. For the example above, five all-α protein structures with α-helices of length = 12 AARs (H) were aligned. Structures B, D and E have a second α-helix with 12 amino acid residues (marked with an “h”–all in bold), and these structures were aligned too, as shown in the bottom three lines. To the left and the right of the PSSE N- and C-terminal ends, respectively, all positions are extended to the 32nd position. Some posts are filled with a “-”, meaning a gap. Those gaps were introduced at the corresponding spots due to a lack of occupation of those loci in selected proteins.

The positional/structural alignment was made considering fixed lengths of α-helices for each of the sizes found in our datamart starting with PSSEs that were a minimum of five amino acid residues long and ending with the maximum encountered value. As we mentioned above, to analyse the nanoenvironment of the α-helices, the observation field was extended by 32 amino acid residues before and after the N- and C-terminal ends (marked with “*”, meaning that any residue might be found at that position). However, those residues might be in any PSSE class, except for the H right before the N-terminal end and after the C-terminal end of the analysed PSSE. However, in cases of missing residues (to complete the desired 32 ones before or after the selected PSSE), the residues were noted by using gaps in these positions: “-” (Fig 4).

### Descriptor selection

We selected 69 descriptors from the extensive list of STING_RDB descriptors and grouped them in seven categories (Table 2). These descriptors describe the characteristics of the nanoenvironment where the α-helices form. In the Supporting Information, we offer an explanation and detailed description of these descriptors.

**Table 2:** List of STING_RDB descriptors used in this work. Although the Supporting Information contains a detailed description, here, we explain some of the acronyms used.

Structural (I)	18. HBMWS * (56)	35. HBMS * (55)
1. Temperature_Factor_CA *	19. HBMWWS *	36. HBMWS * (56)
2. Dihedral_Angle_PHI *	20. HBSS	37. HBMWWS * (57)
3. Dihedral_Angle_PSI *	21. HBSWS	38. HBSS * (58)
4. Dihedral_Chi1*	22. HBSWWS	39. HBSWS (59)
5. Dihedral_Chi2	23. Hydrophobic	40. HBSWWS * (60)
6. Dihedral_Chi3	24. Aromatic	41. Hydrophobic (61)
7. Dihedral_Chi4	25. Ch_attractive *	42. Aromatic (62)
8. Density IFR	26. Ch_repulsive	43. Ch_attractive * (63)
9. Density Internal *	27. Disulfide *	44. Ch_repulsive (64)
10. Space Clash number of clashes *	Unused Contacts (IV)	45. Disulfide * (65)
11. Space Clash percent *	28. Number_Unused_Contact	46. Number_Unused_Contact * (66)
Geometric (II)	Physical Chemical (V)	47. Electrostatic_Potential_at_CA (67)
12. Cross_Link_Order_CA *	29. Electrostatic_Potential_at_CA *	48. Electrostatic_Potential_Average (68)
13. Cross_Pres_Order_CA	30. Electrostatic_Potential_Average	49. Electrostatic_Potential_at_LHA (69)
Contacts (III)	31. Electrostatic_Potential_at_LHA	Others (VII)
14. HBMM *	WNA^(*)^ by Distance and at Surface (VI)	50. Accessible_in_Isolation
15. HBMWM *	32. HBMM * (52)	51. Hydrophobicity_KDI
16. HBMWWM	33. HBMWM * (53)	
17. HBMS *	34. HBMWWM * (54)	

HBMM: main chain to main chain hydrogen bond. HBMWM: main chain to (one H2O) to main chain hydrogen bond. HBMWWM: main chain to (2 H2Os) to main chain hydrogen bond. HBMS: main chain to side chain hydrogen bond. HBMWS: main chain to (one H2O) to side chain hydrogen bond. HBMWWS: main chain to (two H2O) to side chain hydrogen bond. HBSS: means side chain to side chain hydrogen bond. HBSWS: side chain to (one H2O) to side chain hydrogen bond. HBSWWS: side chain to (two H2Os) to side chain hydrogen bond. Ch_attractive means an attractive charge interaction, and Ch_repulsive means a repulsive charge interaction.

(*) The weighted neighbour average (WNA) is calculated by a weighting according to distance among interacting atoms and the accessibility at the surface (Equations 9 and 10, respectively, in the Supporting Information). Hence, the number of WNA descriptors should be counted twice (numbers in ascending order within brackets on the right side of that column). Descriptors whose order numbers are marked with a * are those that passed the radar plot test.

It is not within the scope of this manuscript to discuss in detail the descriptors used here. The readers are welcome to see the description in [40]; in [51]; and at the Sting web site: http://www.cbi.cnptia.embrapa.br/SMS/STINGm/help/MegaHelp_JPD.html

### Data preparation, calculations and presentation

**Fig 5:**
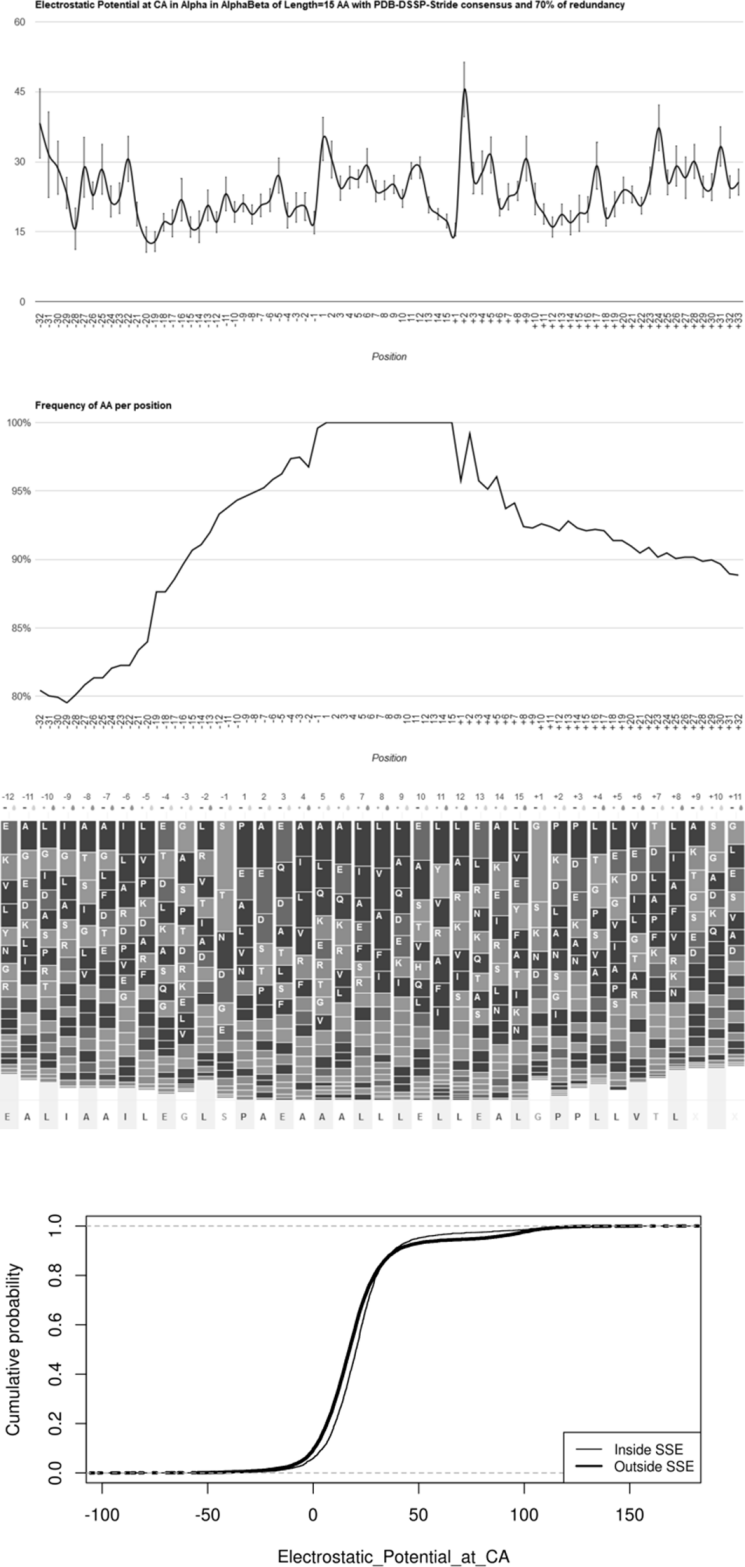
The Protein Secondary Structure Sting Analyzer (PS3A) panels contain four types of plots. In the case shown, 987 α-helices that are 15 amino acid residues long were examined from the datamart in which we removed 70% of the redundancy at the whole protein sequence level, and all instances of α-helices were taken from both all-α and (α+β)+(α/β) proteins. The consensus definition used to determine the presence of an α-helical structure within proteins was the PDB-DSSP-Stride–the most rigorous one. The total number of such proteins is indicated in the Supporting Information in Figure B in S1 File. Plots produced by the PS3A software: A) XY plot for average values (± SEM) for the selected descriptor: electrostatic potential at the α-carbon atom (CA). Negative numbers along the x-axes indicate locations to the left of the N-terminal of the examined/central PSSE, and positive ones follow its C-terminal end. B) The degree of occupancy per AAR position or “reliability”, which is the estimate of how accurately the signal may be observed in A) above. This estimate is only based on how many amino acid residues are present at any location of the positional alignment of the PSSE. The maximum value (100% reliability) is assumed for the ensemble of studied samples along the PSSE. Outside the PSSE, the reliability is usually lower than 100%. C) The sequence logo presents which amino acid type is more frequently found at each positional alignment location–basically indicating the consensus sequence of the PSSE for a selected length (also shown at the bottom part of the logo). The amino acid position numbers (shown on the upper part of the plot) follow the same convention described for A) above. D) The ECDF curve shows how the descriptor average values inside the PSSE region are different from the corresponding values outside the selected PSSE. All of these plots (for each selected PSSE length, type of protein and redundancy level) may be accessed at https://www.ps3a.cbi.cnptia.embrapa.br.

The fifth step for the global pipeline developed in this work was to calculate the average values, standard deviation and standard error of the mean (SEM) for each descriptor listed in Table 2 and across the analysed region, which includes the PSSE and the 32 amino acid residues before its N-terminal and after its C-terminal. We developed the Protein Secondary Structure Sting Analyzer (PS^3^A) software (Fig 5) so that a user could visualize the “signal”/“signature” of the alignment in the form of an XY type of plot (A). Additionally, this software shows the reliability plot (B), the sequence logo (C), and the empirical cumulative distribution function (ECDF) curve (D). The user may access PS^3^A at https://www.ps3a.cbi.cnptia.embrapa.br.

The combination of any of the available 69 STING descriptors (see Table 2) with 18 identified sizes (in DM2_e) for the PSSEs used in this work with two different flavours (a protein with only a single α-helix was called “exclusive”, and a protein with more than one α-helical element was called “nonexclusive”) was in fact a fraction of DM2_ne (only for those lengths found in DM2_e) and resulted in 2484 plots. The sizes of the PSSEs identified were 5–18, 20–21, 30 and 40. At https://www.ps3dv.cbi.cnptia.embrapa.br/, the user may visualize/explore/analyse each of those plots stored in the carousel and save the plots for subsequent use.

## Results

### A constitutive set of parameters defining the PSSE nanoenvironment

**Fig 6:**
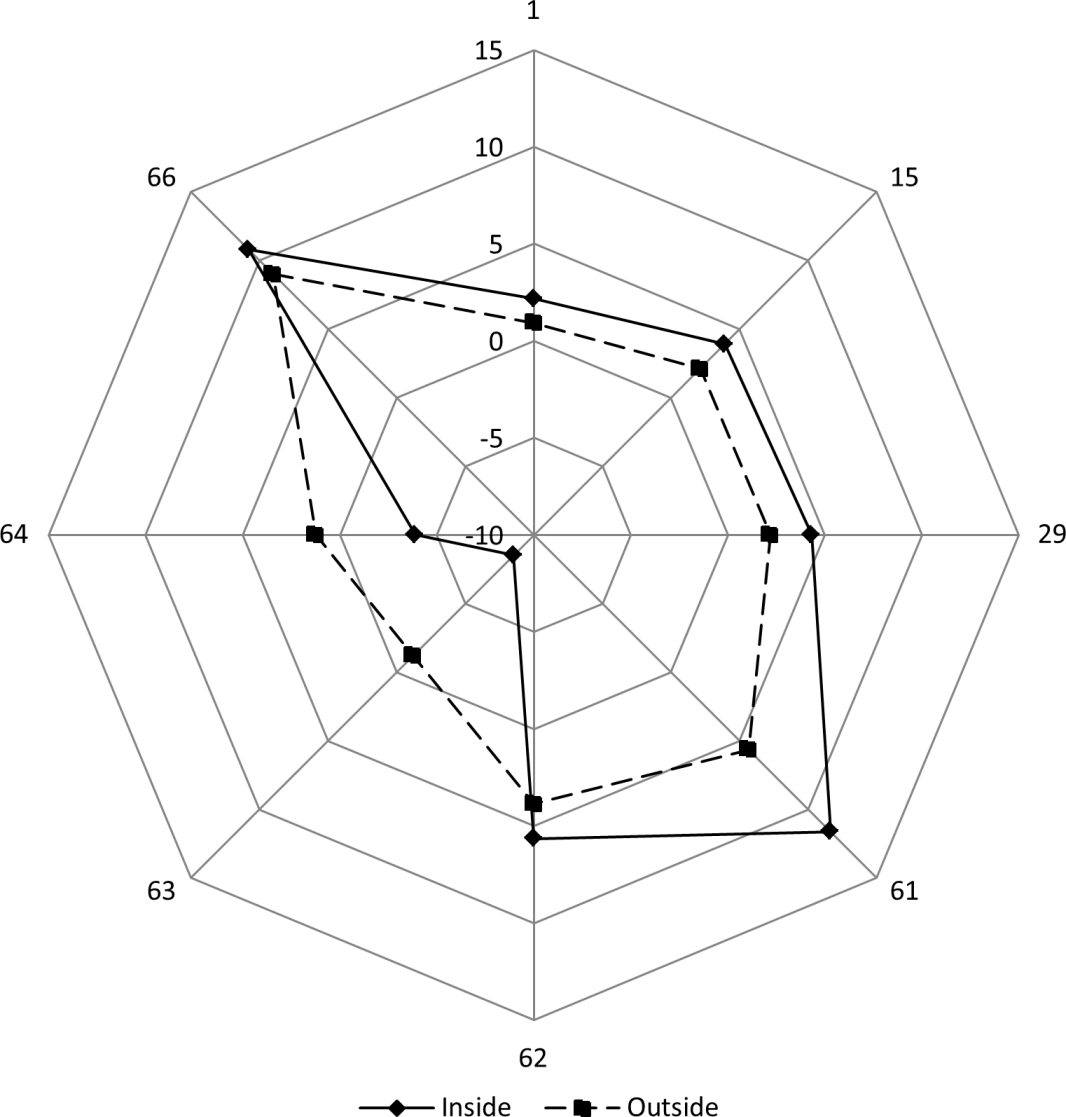
Comparison of the average values of 8 descriptors, normalized (by inverse coefficient of variation) done by dividing the parameter values with the corresponding standard deviation, and calculated for regions inside (17 AAARs) and outside the PSSE. The following descriptors are likely to show the postulated “signal” (the differences between the inside and outside descriptor values per position are higher than 1): 1. Hbmm, 15. Hbmm_WNADist, 29. Hbmm_WNASurf, 61. Number_Unused_Contact_WNADist, 62. Number_Unused_Contact_WNASurf, 63. Dihedral_Angle_PHI, 64. Dihedral_Angle_PSI, 66. Density. The two shadowed descriptors are expected to show differences, as these descriptors are basically part of the definition of the investigated PSSE.

**Fig 7:**
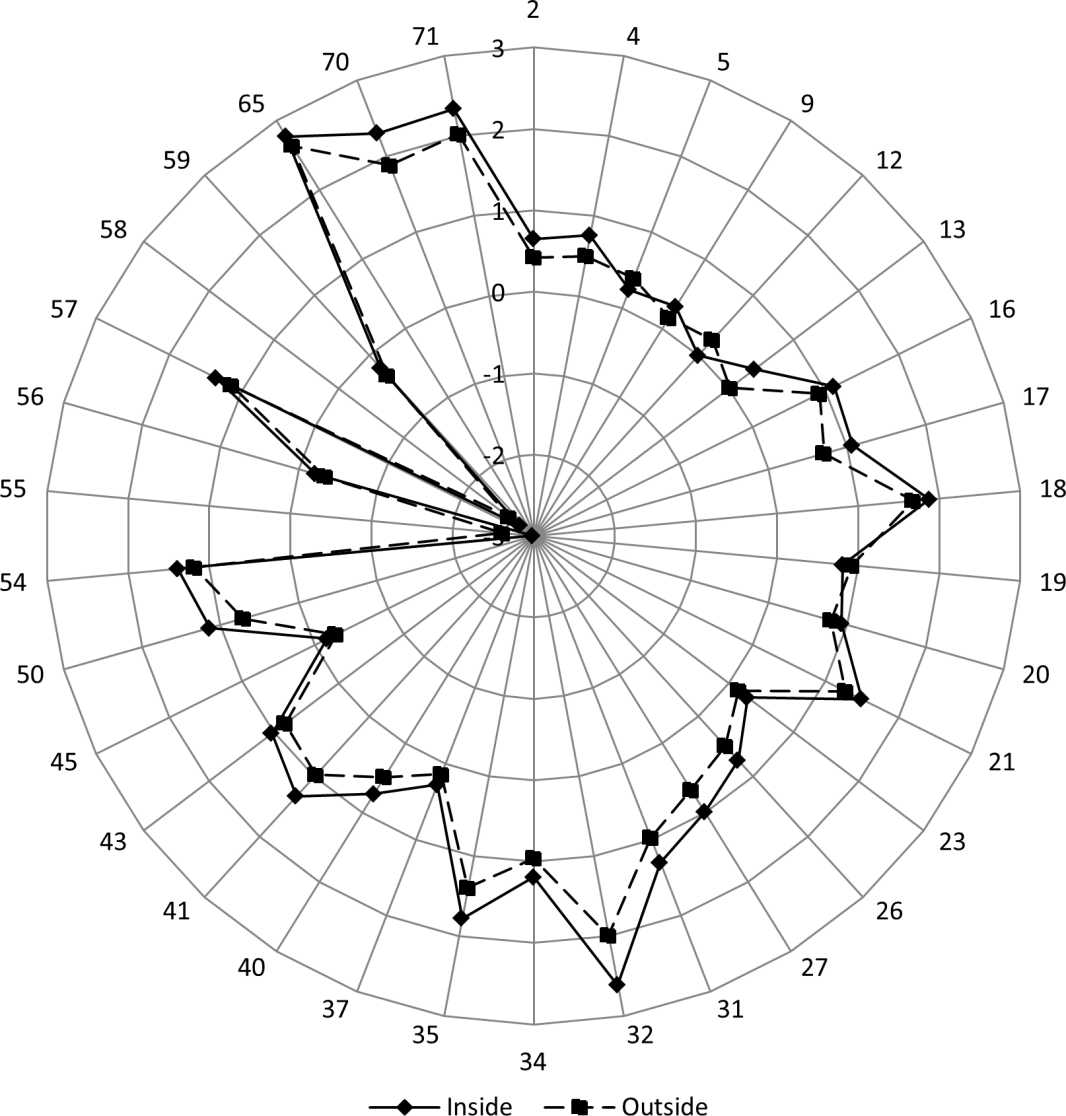
Comparison of the average values of 34 descriptors, normalized (by inverse coefficient of variation) done by dividing the parameter values with corresponding standard deviation, and calculated for regions inside (17 AAARs) and outside the PSSE. The following descriptors are likely to show the postulated “signal” (the differences between the inside and outside descriptor values per position are higher than 0.1 and lower than 1): 2. Hbmwm, 4. Hbms, 5. Hbmws, 9. Hbswws, 12. Disulfide, 13. Ch_attractive, 16.hbmwm_WNADist, 17. Hbmwwm_WNADist, 18. Hbms_WNADist, 19. Hbmws_WNADist, 20. Hbmwws_WNADist, 21. Hbss_WNADist, 23. Hbswws_WNADist, 26. Disulfide_WNADist, 27. ch_attractive_WNADist, 31. Hbmwwm_WNASurf, 32. Hbms_WNASurf, 34. Hbmwwm_WNASurf, 35. Hbss_WNASurf, 37. Hbswws_WNASurf, 40. Disulfide_WNASurf, 41. Ch_attractive_WNASurf, 43. Cross_Link_Order_CA, 45. Dihedral_Chi1, 50. Electrostatic_Potential_at_CA, 54. Electrostatic_Potential_at_CA_WNADist, 55. Electrostatic_Potential_at_LHA_WNADist, 56. Electrostatic_Potential_Average_WNADist, 57. Electrostatic_Potential_at_CA_WNASurf, 58. Electrostatic_Potential_at_LHA_WNASurf, 59. Electrostatic_Potential_Average_WNASurf, 65. Temperature_Factor_CA, 70. SC_Clash, 71. SC_Percent.

It is intuitively clear that a set of parameters rather than just one descriptor (or a couple descriptors) is definitely more suitable for a full description of the nanoenvironment in the context defined above. Figs 6 and 7 demonstrate how 42 parameters (of the 69 selected for this work) differ (in normalized values) inside versus outside the PSSE. In this particular case, we analysed a set of 69 parameters for 28 α+β protein structures containing a single helix of 17 AAR per PSSE and calculated the average and standard deviation values for each parameter. Normalized values were calculated by dividing the average by the standard deviation. This process in fact is normalization by the inverse coefficient of variation, which permits easier reading of the difference between two data sets, which otherwise have very similar values [52]. To demonstrate the extent of the difference in the parameters inside and outside the PSSE, we calculated the difference between the normalized values. From a total of 69 parameters, 34 show a difference (between 0.1 and 1.0) between the average values inside versus outside the helix, and for 8 parameters, this difference is greater than 1, totalling 42 parameters (approximately 60.9% of the set of 69 parameters). Those parameters appear to constitute a statistically significant “signal” (two of examples are depicted at Figure D and Figure E in S1 File in Supporting Information) for defining the α-helical nanoenvironment. The coverage for exclusive helices is rather broad, the same number of SSE signals containing 42 descriptors remains nearly the same for PSSE sizes of 11 to 40 (decreasing to 38 for helix size 6). On the other hand, for nonexclusive helices, the coverage decreases from 54 descriptors for helix size 6, to 42 descriptors for helix size 40.

### Multiple variable analyses

**Fig 8:**
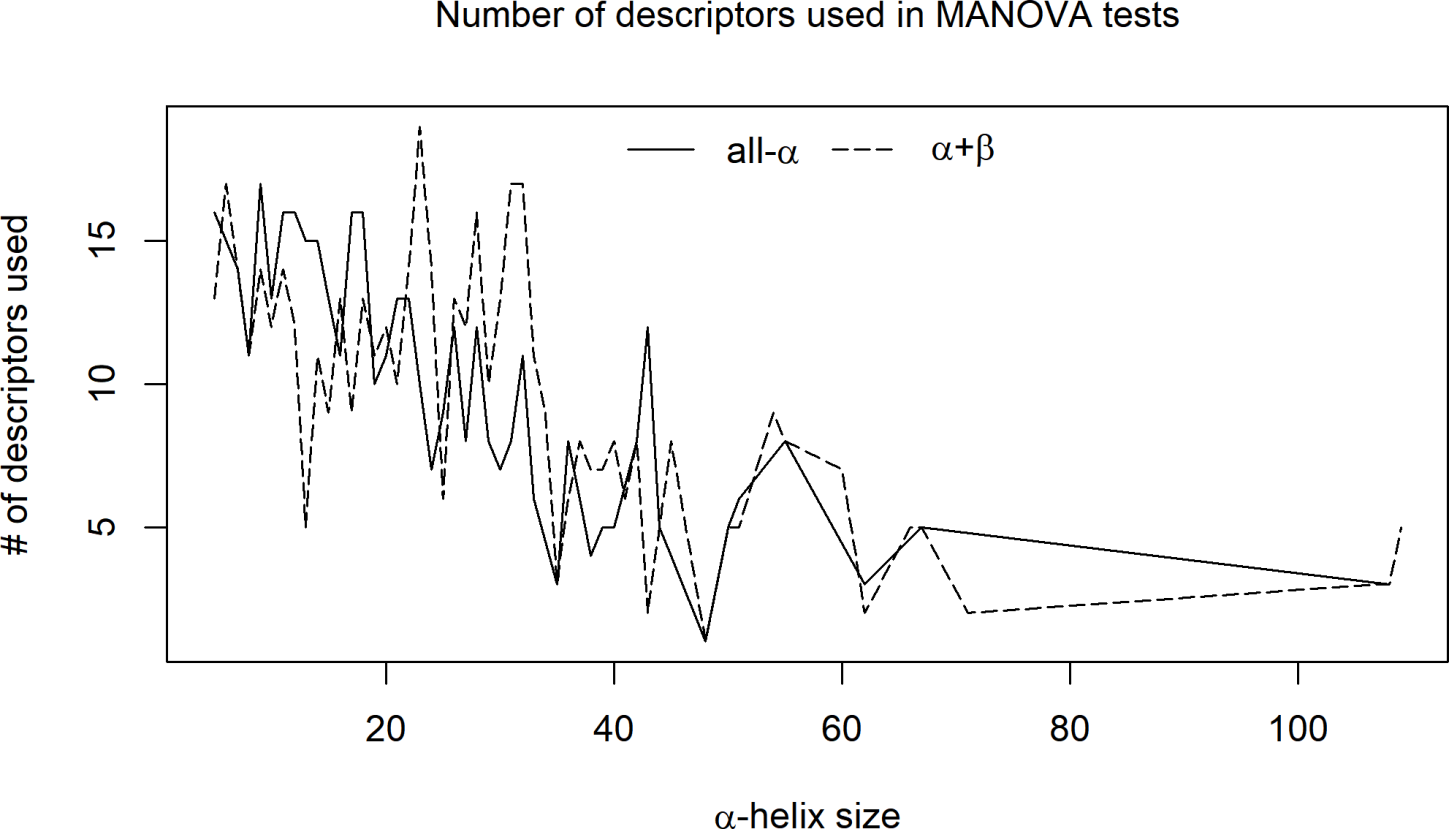
Variation in the number of descriptors that passed both the normal distribution test and the no mutual correlation test for different helix sizes.

In the multivariate analysis of variance (MANOVA), it was necessary to first remove those descriptors that did not obey a normal distribution. In addition, linearly correlated descriptors (defined after using an R script and default threshold/cutoff of 0.9) were also eliminated. As a result, in almost all of the tests, the number of descriptors actually used in the MANOVA was lower than the number of descriptors provided to the test input. As described in Fig 8, as the size of the SSE increases, the number of descriptors that passed both the normal distribution test and the no mutual correlation test decreases. Consequently, the MANOVA test had less descriptors: approximately 10 to 15 for helix sizes up to 25 AARs and then decreasing to approximately 5 descriptors for sizes of 40 AARs and above. The lowest number of descriptors used in this test was 3 (for a helix size of 108 AARs). The average number of descriptors for all helix sizes was 10. For all helix sizes, the four applied MANOVA tests showed p-values lower then 1x10^-6^ in at least 83% of the cases and lower than 1x10^-3^ in at least 95% of the cases. Such results clearly support our initial hypothesis that a selected number of qualified descriptors may fully identify and describe the internal nanoenvironment of the protein region containing the helical structure.

**Fig 9:**
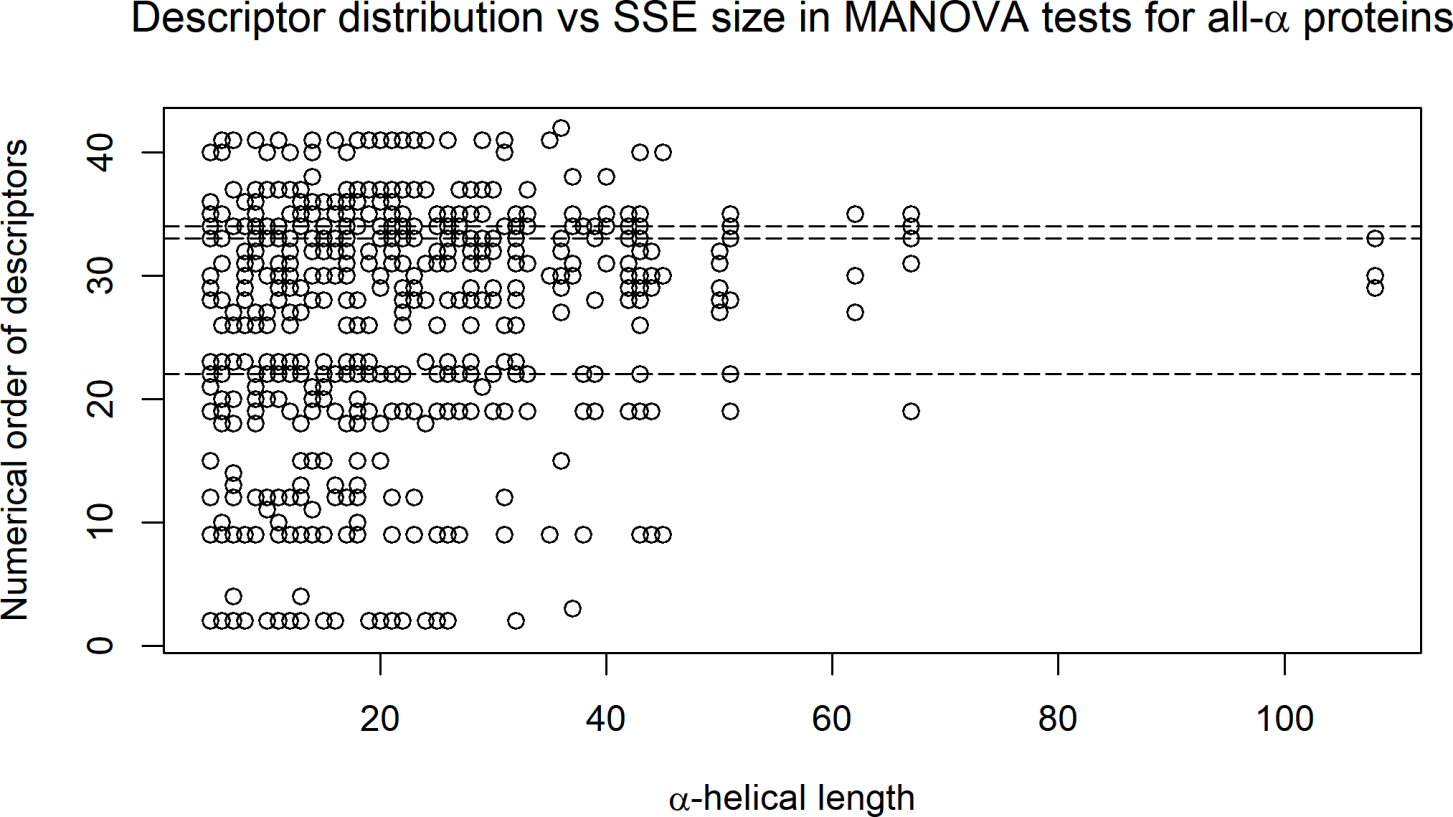
Representation of 42 different descriptors used for the MANOVA input and then filtered by a double test: A normal distribution of data and a lack of mutual correlation. The points, which are plotted for each helical size (x-axes), represent those descriptors used by MANOVA for that particular size. The 42 descriptors found on the y-axes are as follows: 1. Hbmm, 2. Hbmwm, 3. Hbms, 4. Hbmws, 5. Hbswws, 6. Disulfide, 7. Ch_attractive, 8. Hbmm_WNADist, 9. hbmwm_WNADist, 10. Hbmwwm_WNADist, 11. Hbms_WNADist, 12. Hbmws_WNADist, 13. Hbmwws_WNADist, 14. Hbss_WNADist, 15. Hbswws_WNADist, 16. Disulfide_WNADist, 17. ch_attractive_WNADist, 18. Hbmm_WNASurf, 19. Hbmwm_WNASurf, 20. Hbmwwm_WNASurf, 21. Hbms_WNASurf, 22. Hbss_WNASurf, 23. Hbswws_WNASurf, 24. Disulfide_WNASurf, 25. Ch_attractive_WNASurf, 26. Electrostatic_Potential_at_CA, 27. Electrostatic_Potential_at_CA_WNADist, 28. Electrostatic_Potential_at_LHA_WNADist, 29. Electrostatic_Potential_Average_WNADist, 30. Electrostatic_Potential_at_CA_WNASurf, 31. Electrostatic_Potential_at_LHA_WNASurf, 32. Electrostatic_Potential_Average_WNASurf, 33. Number_Unused_Contact_WNADist, 34. Number_Unused_Contact_WNASurf, 35. Cross_Link_Order_CA, 36. Dihedral_Chi1, 37. Dihedral_Angle_PHI, 38. Dihedral_Angle_PSI, 39. Temperature_Factor_CA, 40. Internal_CA_3, 41. Clash, 42. Percent. Finally, the three most frequently plotted descriptors are as follows (designated by the three horizontal dashed lines, from top to bottom): 1. Electrostatic_Potential_Average_WNASurf (order number: 32) ≥ (30, 65%), 2. Number_Unused_Contact_WNADist (order number: 33) ≥ (30, 67%) and 3. Hbms_WNASurf (order number: 21) ≥ (26, 58%).

The ten most frequent descriptors used by MANOVA (after the descriptors passed the double test on the input) were as follows (see also Fig 9): 1. Electrostatic_Potential_Average_WNASurf (30, 65%), 2. Number_Unused_Contact_WNADist (30, 65%), 3. Hbms_WNASurf (26, 57%), 4. Hbmm_WNASurf (25, 54%), 5. Number_Unused_Contact_WNASurf (25, 54%), 6. Hbmm_WNADist (24, 52%), 7 Electrostatic_Potential_at_CA_WNADist (23, 50%), 8. Electrostatic_Potential_Average_WNADist (22, 48%), 9. Dihedral_Chi1 (20, 43%), and 10. Electrostatic_Potential_at_CA_WNASurf (20, 43%). The numbers within parentheses represent the total number of appearances among the descriptors used and the percentage of the total number of helix sizes where that particular descriptor was used by MANOVA, respectively.

The situation in α+β protein structures is similar; however, the 10 most frequently found descriptors are as follows: 1. Number_Unused_Contact_WNADist (37, 69%), 2. Electrostatic_Potential_at_LHA_WNADist (30, 56%), 3. Electrostatic_Potential_Average_WNASurf (29, 54%), 4. Number_Unused_Contact_WNASurf (29, 54%), 5. Electrostatic_Potential_at_LHA_WNASurf (28, 52%), 6. Electrostatic_Potential_at_CA_WNADist (25, 46%), 7. Electrostatic_Potential_at_CA_WNASurf (24, 44%), 8. Hbms_WNASurf (21, 39%), 9. Cross_Link_Order_CA (20, 37%), 10. Dihedral_Chi1 (20, 37%). The lowest number of descriptors used in this test was 2 (for a helix size of 109 AARs). The average number of descriptors for all helix sizes in α+β proteins was 10 again.

For α+β proteins and all helix sizes, the four applied MANOVA tests showed p-values lower then 1x10^-6^ in at least 85% of the cases and lower than 1x10^-3^ in at least 92% of the cases. Once again, the null hypothesis was easily ruled out.

We conducted one more test in order to estimate the power of the most frequently used descriptors (listed above) to distinguish the nanoenvironment of an α-helix from the non-helical environments. In the case of all-α proteins, the 10 most frequently used descriptors would give us approximately 70% coverage and p-values lower than 10^−3^ in more than 90% of the cases. The first four most used descriptors gave 63% coverage and p-values less than 10^−3^ in more than 83% of the cases. For nonexclusive α-helices, using the 10 corresponding most frequently used descriptors yielded an 84% coverage and p-value less than 10^−3^ in more than 96% of the cases. The first 5 of the aforementioned descriptors gave 55% coverage and p-values less than 10^−3^ in more than 73% of the cases.

### Moving/sliding window of the average values for a selected descriptor

**Fig 10:**
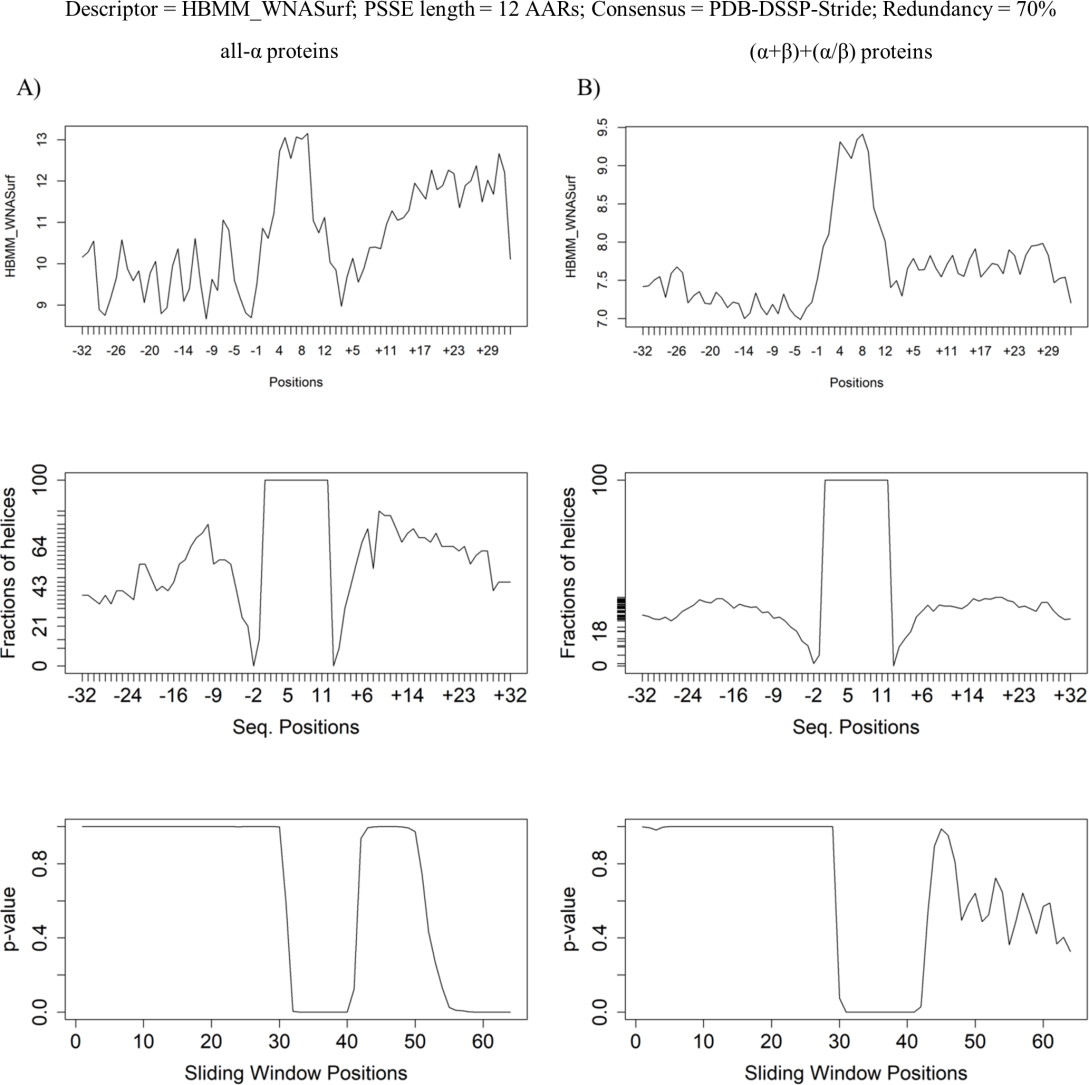
Composite graphs showing the following: Descriptor variation along the regions before, at and after the analysed PSSE; the reliability value (or % of helical structure at each loci) and the p-value for the descriptor: Number of contacts, type “HBMM”. Data are drawn from the datamart containing PSSEs of length = 12 AARs; the consensus definition of a helix element is from “PDB-DSSP-Stride”, and the redundancy is 70% similarity at the sequence level.

Fig 10 is a compilation of plots, and each set (A-B) has three different graphs. The first graph shows the variation in the average value of the descriptor: in this case–the number of contacts of HBMM type(53), weighted by the distance measured over the surface to which the AAR belongs. Sequence redundancy was removed at the 70% similarity level, revealing 24 helices in the all-α structures (on the left side of the figure). The same level of redundancy was set for the α-helices in the (α+β) + (α/β) structures, revealing 203 helices, which also had 12 AAR (on the right side of the figure). The average descriptor value is shown for each location of the positional alignment of α-helix size. Along the horizontal axis, “negative” positions (“‒”) correspond to the amino acid residues located before the PSSE N-terminal end, while “positive” positions (“+”) correspond to the amino acid residues after the PSSE C-terminal end. The second graph shows the degree of occupancy per position of the encountered amino acid residues (of any type) that belong to the α-helical structure at any particular position presented in that plot (where that number is 100 along the PSSE location itself and lower is lower to the left and right of the PSSE). Therefore, the reliability is 100% along the examined PSSE. In addition, some residues might be missing (to the left and the right of the analysed PSSE) as the stretch might not reach the desired limits of the N-terminal -32 to C-terminal +32 residues. The temporary decrease immediately before and immediately after the examined PSSE, followed by an increase in reliability, is because the PSSE limits must be delimited by AARs, which definitely may not belong to a helix (that being the definition of PSSE limit). Further from the N- and C-termini, the presence of other α-helices is possible, and the reliability therefore oscillates until positions -32 and +32. The reduction in reliability at the very N-terminal and C-terminal ends of the extended PSSE region results from the surge in gaps, which become ever more frequent and which we introduce in such cases, usually at the positions discussed here where the original sequence does not reach the desired limit position (±32). The third plot represents the data resulting from the sliding window test. Namely, using an R script, the descriptor values were grouped in the window with the same length of the selected PSSE and that window was run (slide) from the left to the right along the positional alignment of the α-helical element and its extended region. Additionally, other sliding windows of varying length, from one AAR to double the PSSE size, were also made to slide along the same path. This experiment proved that it is possible to identify the PSSE region by a sliding window test, preferably using a window with the minimum possible length, as proven by the results depicted in Figure H in S1 File. Finally, Student’s t-test was applied to data that were divided into two sets: data for AARs from within and from outside the sliding window range. As shown in Fig 10, the p-value from Student’s t-test approaches zero exactly within the region where the sliding window matches the PSSE position. That observation corroborates the hypothesis we postulated at the beginning of this work. In general, if, as in the third plot of Fig 10A and 10B, the p-value demonstrates such a clear variation, then the ensemble inside the sliding window at a selected position is significantly different from the ensemble outside that window region. Although some regions other than the central PSSE regions appear to have a p-value approaching zero, these regions are mostly outside the PSSE where the p-value exhibits a different/specific behaviour compared to the behaviour observed for the PSSE flanking regions. Even though the PDB does not contain any single helix protein structure with a long enough sequence to satisfy the conditions of our experiment, which would create the situation where we would have flanking regions with no perturbation from other helical structures or turn structures, it is quite clear from our plots that such signals / perturbations do exist in the experimental setup. This possibility clearly might explain partially why we have p-values fluctuating in the flanking regions.

After being convinced of the validity of the initial hypothesis of this work (valid for multiple descriptors in multivariate analysis), we needed to closely examine some specific SSE sizes and selected parameters/descriptors, aiming to identify the upper limit for coverage in the case of using only a single descriptor.

The Kolmogorov-Smirnov test was applied in order to statistically confirm the previous visual observations for some of the data analysed here regarding the “existence” of a nanoenvironment-specific single descriptor “signal”. A total of 46 different helical lengths (sizes) (DM1) were subjected to statistical examination using one out of available 69 descriptors (see Table 2) for a total of 3165 available tests (9 tests were excluded because they showed an input data problem). A total of 125 tests have a p-value equal to or less than 1x10^-6^ (3.9%) exactly over the region fully matching the extent/position of the examined α-helix. Additionally, 426 tests show p-values along the encountered α-helices equal to or less than 1x10^-3^ (13.5%). These results indicate that the analysed tests show p-values compatible with the conclusion that the helical region (in terms of nanoenvironment) is significantly different from the regions outside the helix. Such relatively low performance of the success indicator becomes more favourable for the postulated existence of a “signal” for α-helices found in (α+β)+(α/β) proteins: a total of 54 different helical sizes (DM2) were subjected to statistical examination using the same 69 descriptors, for a total of 3704 available tests (22 tests were excluded because they showed an input data problem). For this situation, a total of 766 tests have a p-value equal to or less than 1x10^-3^ (20.7%) and exactly over the region fully matching the extent/position of the examined α-helix, and 298 tests have a p-value smaller than 1x10^-6^ (8%). A more detailed analysis (Table 3) identified very clear-cut situations in which the statistics indicate the existence of a nanoenvironment “signal” in approximately one fifth of the abovementioned cases. In addition, as explained in the “Hypothesis verification section”, when the p-values are close to 0, as in the two mentioned sample marts, then we may reject the H0 hypothesis (which claims that the two datasets–the data from inside the PSSE compared to outside the PSSE–are statistically not distinguishable from each other). The remaining approximately 80% of the studied cases, do not have such low p-values within the region of the analysed PSSE. However, this finding was expected as there is no such unique parameter that has the power to singlehandedly fully describe the nEoαH.

**Table 3:** The KS test applied for sliding windows in all size helices using single parameter analysis.

Datamarts	p-value ≤ 1x10^-6^	1x10^-6^> p-value ≤ 1x10^-4^	1x10^-4^ > p-value ≤ 1x10^-2^	p-value>1x10^-2^
**all-α exclusive**	1,1%	2,4%	7,6%	88,9%
**all-α nonexclusive**	3,9%	4,5%	13,1%	78,5%
**α+β exclusive**	2,9%	3,9%	10,5%	82,7%
**α+β nonexclusive**	8,1%	6,8%	14,3%	70,8%

In conclusion, most of the statistical analysis points towards the possibility of clearly distinguishing a helical nanoenvironment from the environment of the region outside the helix. An apparent upper limit is clearly seen for the usage of a single descriptor. On the other hand, the number of descriptors in multivariate analysis comfortably points to a coverage level greater than 90%.

### Single descriptor “signature” comparison: The case of two different types of PSSE

**Fig 11:**
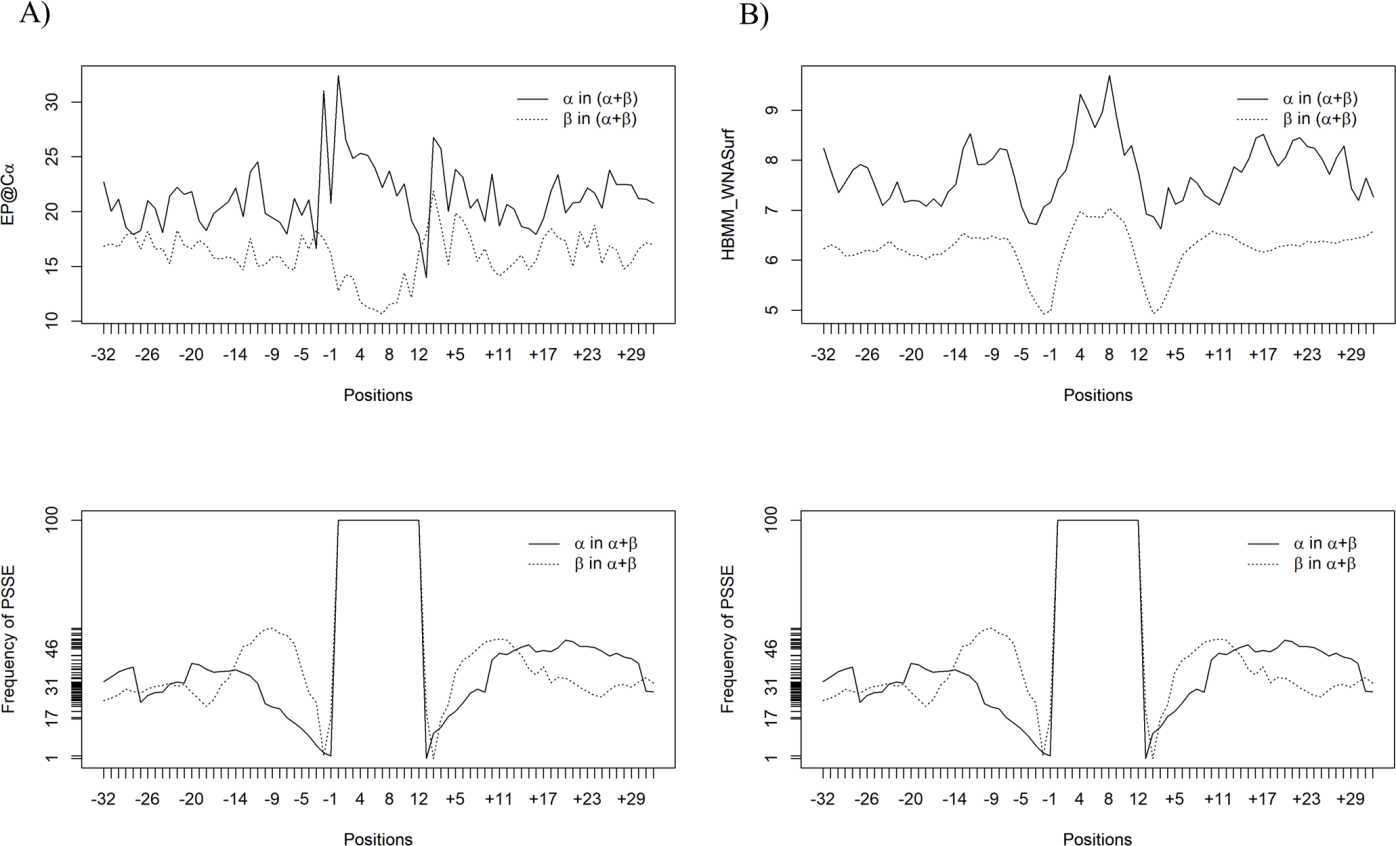
Differences in the variation behaviour of two selected descriptors around α-helices (solid lines) and β-strands (dotted lines). The plots above present the behaviour of the A) EP@Cα average values for 1811 α-helices in (α+β)+(α/β) proteins and 7773 β-strands in (α+β)+(α/β) proteins. B) HBMM_WNASurf average values for α-helices in (α+β)+(α/β) proteins and β-strands in (α+β)+(α/β) proteins. The average number of this contact type is higher in and around α-helices than in and around β-strands. As shown, there are clear differences in signal pattern in the cases presented in A and B.

To demonstrate how representative the analysis we presented so far is, it is also necessary to verify the behaviour for a single descriptor value for an entirely different PSSE–the “β-strand”. If our hypothesis holds, then the same principles should hold as well, while the signal/signature might be different in shape, content (type of descriptor), and intensity. A comparison between the corresponding datasets for α-helical regions against β-strand regions in (α+β)+(α/β) proteins demonstrated that there is a convincing difference in the PSSE “signal” types describing their respective PSSE regions (the compilation for complete β-strand analysis is still under construction and will be published separately). Fig 11 presents the data pointing to the difference between the nanoenvironments for α-helices and β-strand regions. The descriptors and datasets compared were as follows: A) (on the left side of this figure panel) EP@Cα, and B) (on the right side of this figure panel) the number of contacts of HBMM_WNASurf type, in 1657 α-helices in (α+β)+(α/β) proteins against 20790 β-strands in (α+β)+(α/β) proteins chains. The comparison between the EP@Cα moving average values for α-helices in (α+β)+(α/β) proteins and β-strands in (α+β)+(α/β) proteins shows that while the PSSE “signal” resembles the letter “N” in the case of α-helices, at the same time, the “signal” for β-strands resembles the letter “U” more. The HBMM_WNASurf average values for α-helices in (α+β)+(α/β) proteins and β-strands in (α+β)+(α/β) proteins are higher for α-helices than for β-strands.

### Knowledge of the PSSE nanoenvironment applied to quality assessment of protein structures: The case of predicted vs experimentally obtained 3D structures

**Fig 12:**
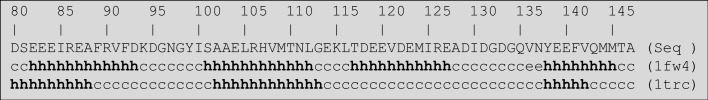
The very same PSSE defined in two protein structures: 1fw4.pdb, with a resolution of 1.7 Å (top sequence), and 1trc.pdb, with a resolution of 3.6 Å (bottom sequence). The 1FW4 structure has one extra α-helix. Both structures have identical AAR sequences. The region starting at residue #117 and ending at residue #127 was used in our experiment as this region has the most obvious discrepancy in SSE assignment.

To demonstrate one example of a practical use of this knowledge and the potential value of analysing the collected data in everyday situations for computational structural biologists, in this section we compared two X-ray structures: 1fw4.pdb, with a resolution of 1.7 Å and an R-factor of 0.220; and a structure with an identical AAR sequence: 1trc.pdb, with a resolution of 3.6 Å and an R-factor of 0.257. The intention here is to evaluate whether the experimentally observed electron densities produce good model structures as judged by the PSSE nanoenvironment “signals”. For the case of a structure that does not have a good resolution/R-free factor, this evaluation is crucial in order to improve the final model; in the case of a structure obtained with good resolution, it is important to know whether the positioning of the PSSE is corroborated by a PS^3^A check-up. If successful, we might then suggest that users employ PS^3^A as a tool to evaluate and guide improvements in the modelled structures with regard to the type and positioning of PSSEs. Fig 12 shows the sequence of both structures and their PSSE definitions. The letter code used is as follows: “h” = α-helices, “c” = coil, and “e” = β-strands.

**Fig 13:**
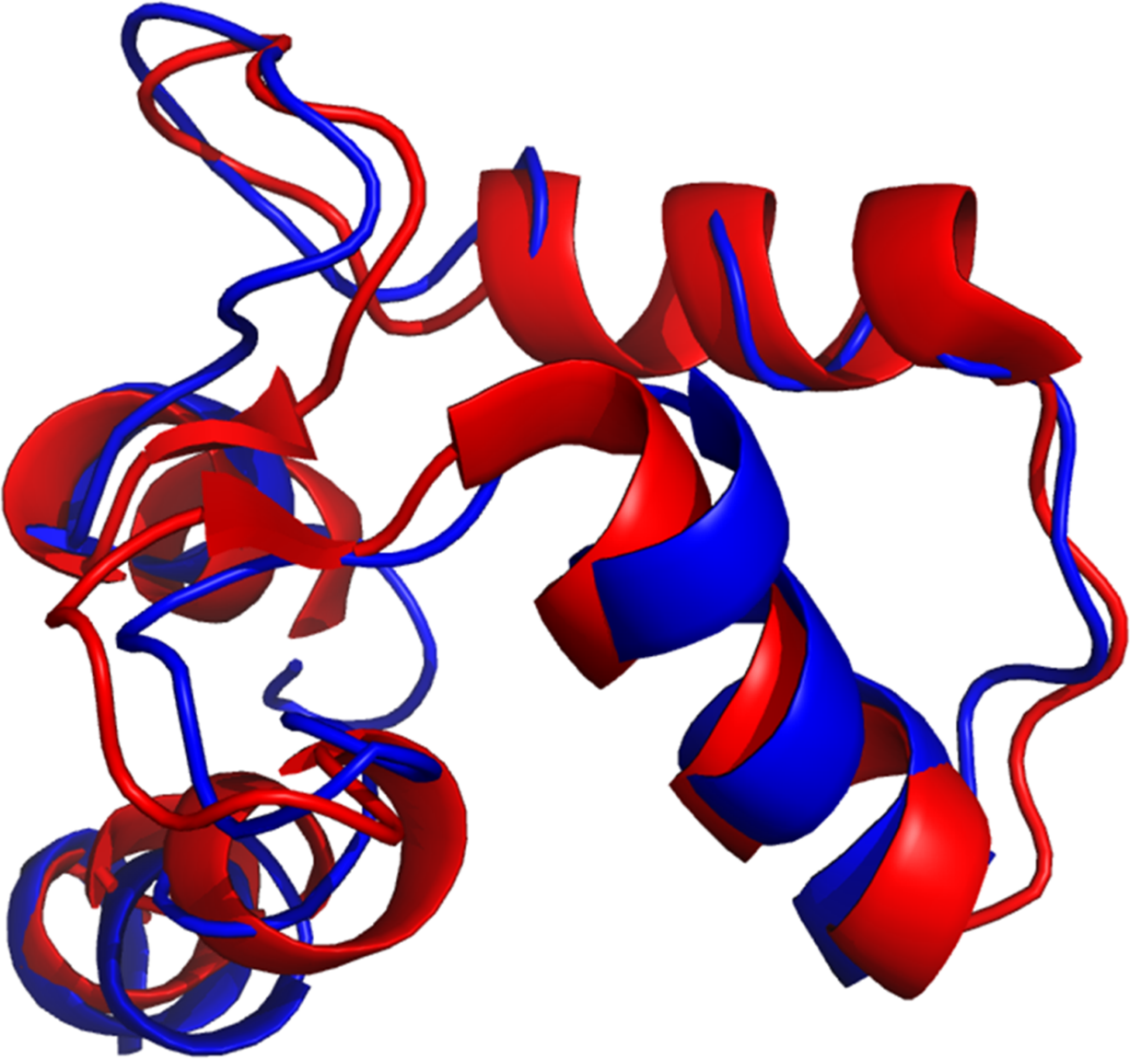
The superposition of two identical proteins whose structures were solved at two very different resolutions. The 1fw4.pdb (red ribbon) structure has a 1.7 Å resolution and 1trc.pdb (blue ribbon), 3.6 Å. Both structures have the very same amino acid sequence, but 1trc.pdb is an older structure, and its low resolution (3.6 Å) causes some errors in the α-helix definition and positioning. The region between 117 and 127 AAR, at the top right, demonstrate that in both cases there is a helical element there but the lower resolution structure does not have a corresponding assignment for it.

Fig 13 demonstrates the structural alignment of the well-resolved structure—1fw4.pdb (red ribbon) versus the poorly resolved structure—1trc.pdb (blue ribbon). Although the poorly resolved structure does not present an α-helix in the analysed area (117–127) because its resolution is lower, our methodology proves that there is an α-helix at that position, as is observed and confirmed in the better resolved structure.

Calculation of p-value in multivariate analysis for the regions inside versus outside the helical structure (inside being from 117th to 127th AAR) returns values of lower than 1x10^-6^ for both structures. This result means that the assignment of the PSSE in the lower resolution structure failed to recognize the presence of a helix in considered region.

From those two very simple experiments, one may clearly conclude that the concept of a nanoenvironment specific for structural and functional regions is a very applicable concept (including the case here studied), and this concept could be applied to quality assessments of protein structures. One should, however be very careful if selecting a single descriptor to be used for such tests, as we have previously shown that a single parameter has only a limited coverage. Multidescriptor vectors are definitely suggested for broader coverage.

## Discussion

At the pathway from the amino acid sequence to the final 3D structure, the secondary structure elements of proteins are constructed, and one of the problems currently facing molecular biologists is understanding how the protein initiates, nucleates and maintains the PSSEs during folding and understanding the final nanoenvironment of both the PSSEs and the amino acid residues in their surroundings.

This paper is the result of extensive work selecting, grouping, preparing and treating data on the nanoenvironment where PSSEs, specifically α-helices, are embedded. The hypothesis that motivated our work was the possibility of the existence of a PSSE “signal,” i.e., a visually and statistically detectable perturbation in the value of selected descriptor(s) hinting at particular characteristics of the nanoenvironment of each type of secondary structure element. The concept of a nanoenvironment was already explored earlier with successful results reported for protein-protein interfaces and enzyme catalytic sites. This work employed the same idea and applied the concept to the case of the PSSE nanoenvironment.

The individual plots in Fig 11 demonstrate a different behaviour inside the PSSE for the “signal” of α-helices, which is not the same as the “signal” of β-strands. However, considering that the nanoenvironment is not fully defined by a unique/single descriptor, a different approach was necessary to confirm the hypothesis of the existence of a “signal” inside a specific PSSE. The application of multivariate analysis of variance (MANOVA) to the same dataset confirmed the existence of a “signal” for α-helices. Based on these tests, we conclude that a set of specific parameters, such as contacts, physical-chemical, geometrical and structural descriptors, describes a nanoenvironment, in this case, the nanoenvironment of α-helices. Three descriptor categories were found to be among the most frequently used for nEoαH identification: Number_Unused_Contacts, Electrostatic_Potential and number of contacts of Hbms type, meaning that the potential for forming contacts, the number of hydrogen bonds (of main chain to side chain type) and the electrostatic potential of the involved AARs are crucial descriptors of the nEoαH.

The next step to continue our work is to confirm the hypothesis for β-strands, turns, and coils. Preliminary tests indicated that the hypothesis would be confirmed in the same way as our confirmation for α-helices.

## Supporting information

S1 File (DOCX)
