# Supplementary material for: Study of specific nanoenvironments containing α-helices in all-α and (α+β)+(α/β) proteins
Source: PLoS One. 2018 Jul 10;13(7):e0200018. doi: 10.1371/journal.pone.0200018 (PMC6039001; doi:10.1371/journal.pone.0200018)
Supplement: S1 File — (DOCX) [file pone.0200018.s001.docx]

# Supporting Information

## Number of chains for each PSSE compilation

Figure A shows the number of chains found in the PDB for all-α proteins and α-helices in (α+β)+(α/β) proteins according to the PDB, DSSP and Stride definitions.


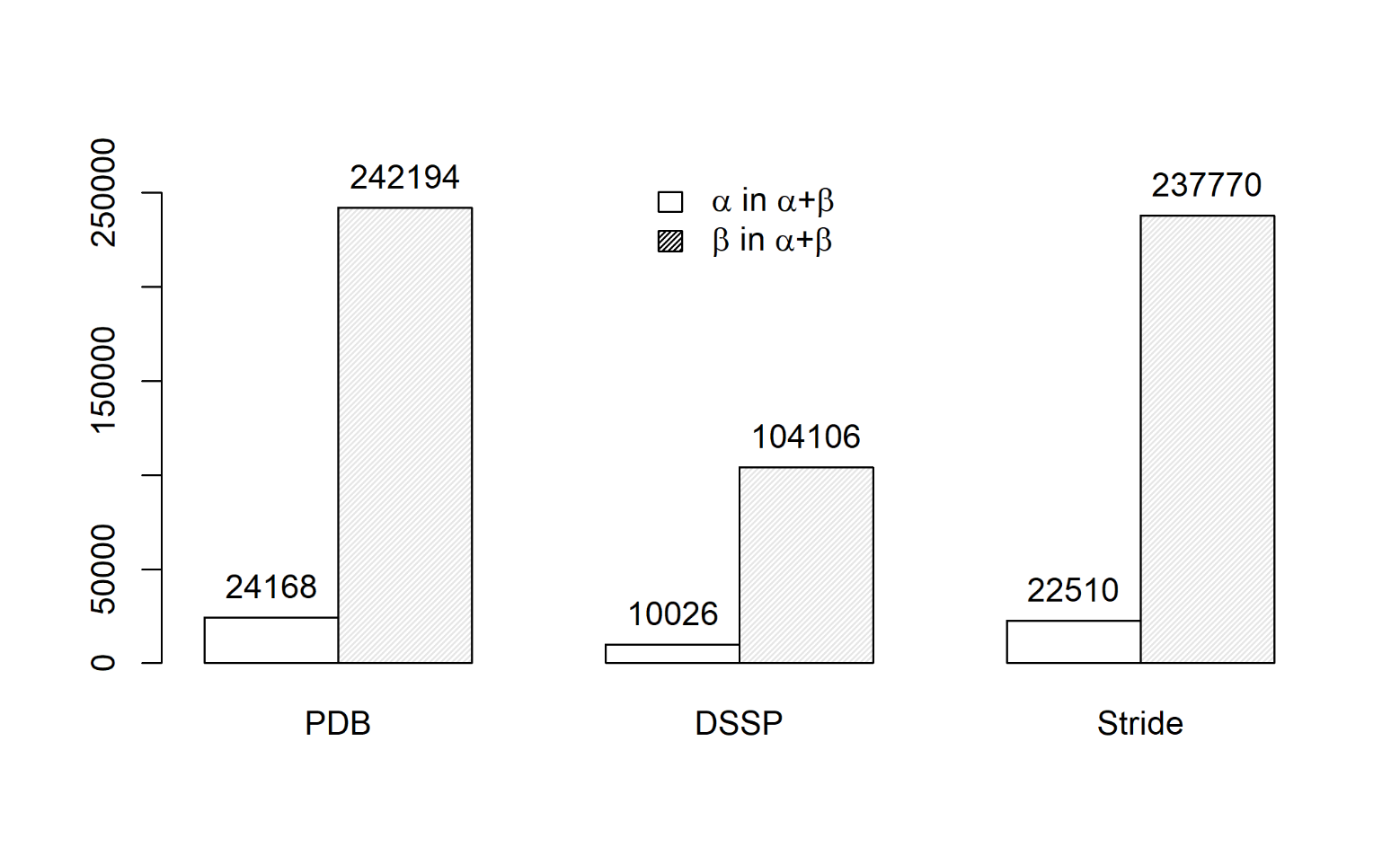


Figure A. Number of chains in the protein structures with α-helices according to PDB, DSSP, and Stride definitions. The Stride algorithm is more likely to define a region as an α-helix than the DSSP algorithm.

As explained in the Materials and Methods section, the whole sequence redundancy was eliminated at the 95%, 70% or 50% similarity level using the CD-HIT software. Figure B shows the number of chains in all-α and in (α+β)+(α/β) proteins after the redundancy removal procedure was completed.


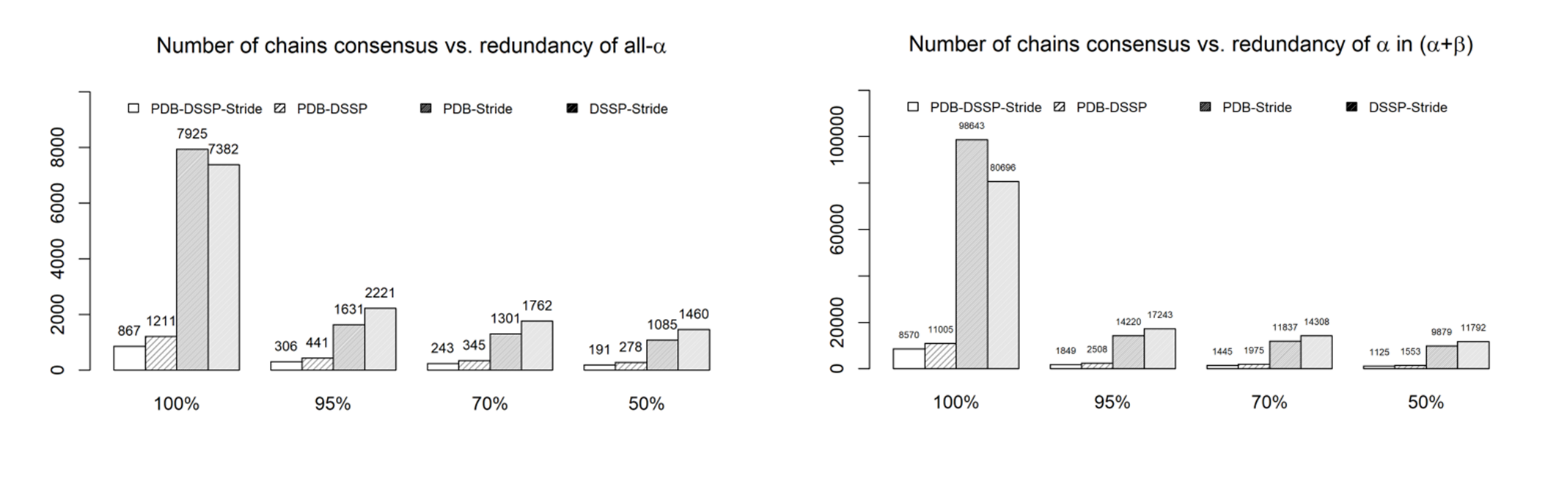


Figure B. Number of chains according to selected PSSE consensus definitions vs sequence redundancy level for all-α proteins (left) and for α-helices in (α+β)+(α/β) proteins (right).

Figure C shows the number of chains grouped by the PSSE consensus definition (PDB, DSSP and Stride). The PDB algorithm has a more restrictive definition for the presence of an α-helix, while the Stride algorithm is less rigorous with such filtration.


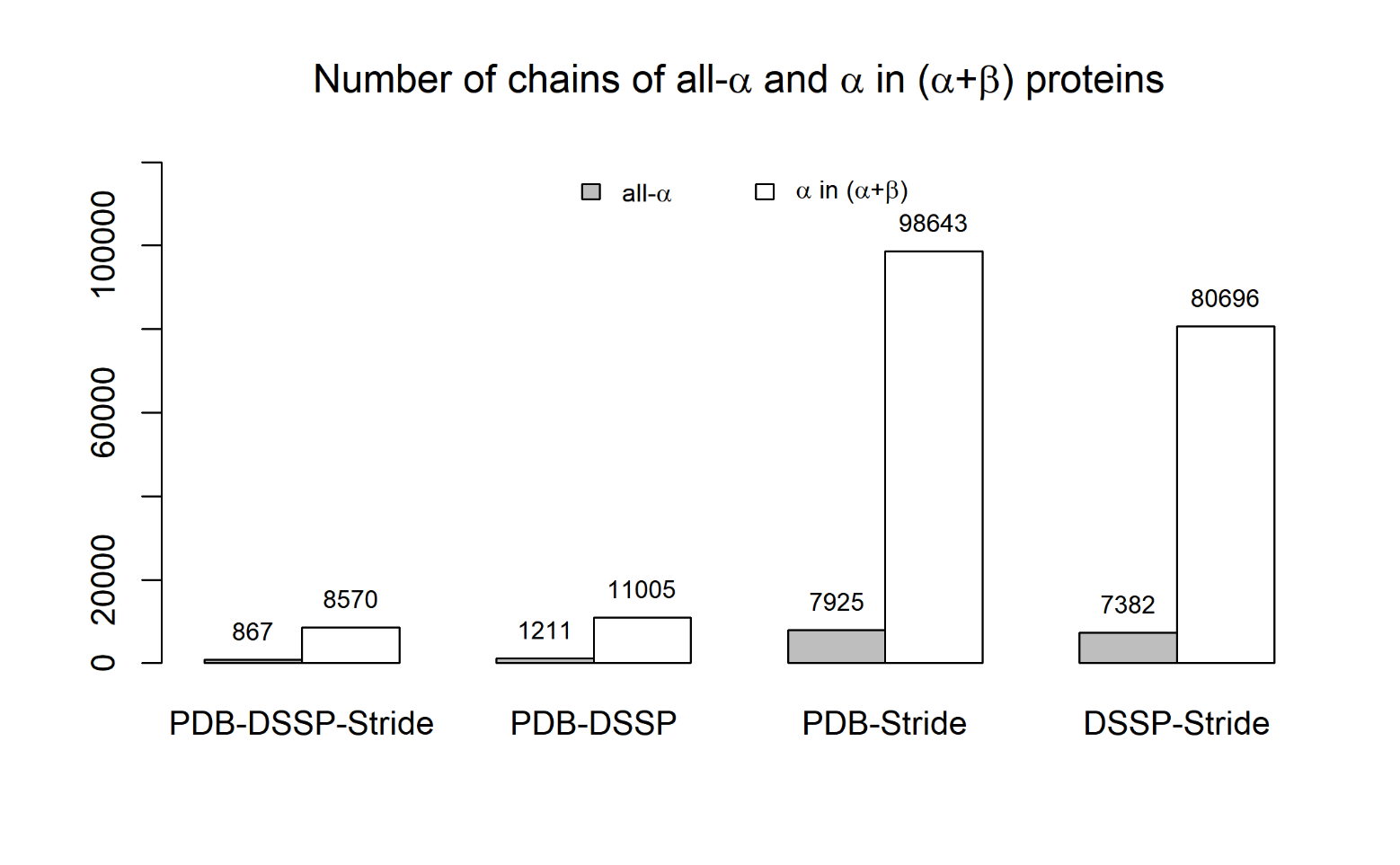


Figure C. Number of chains in all-α proteins and α-helices in (α+β)+(α/β) proteins after the positional alignment, according to the PSSE consensus definition (combinations of the PDB, DSSP and Stride definitions were used).

## Signals/signatures encountered in all-α proteins and in the α-helices in (α+β)+(α/β) proteins with PDB-DSSP, PDB-Stride, and DSSP-Stride PSSE consensus definition

Figure D and Figure E present the “signal”/“signature” found with the PDB-DSSP PSSE consensus definition for HBMM and EP@Cα descriptors in all-α proteins (left) and in the α-helices of (α+β)+(α/β) proteins (right), with other parameters designated in the figure legend.


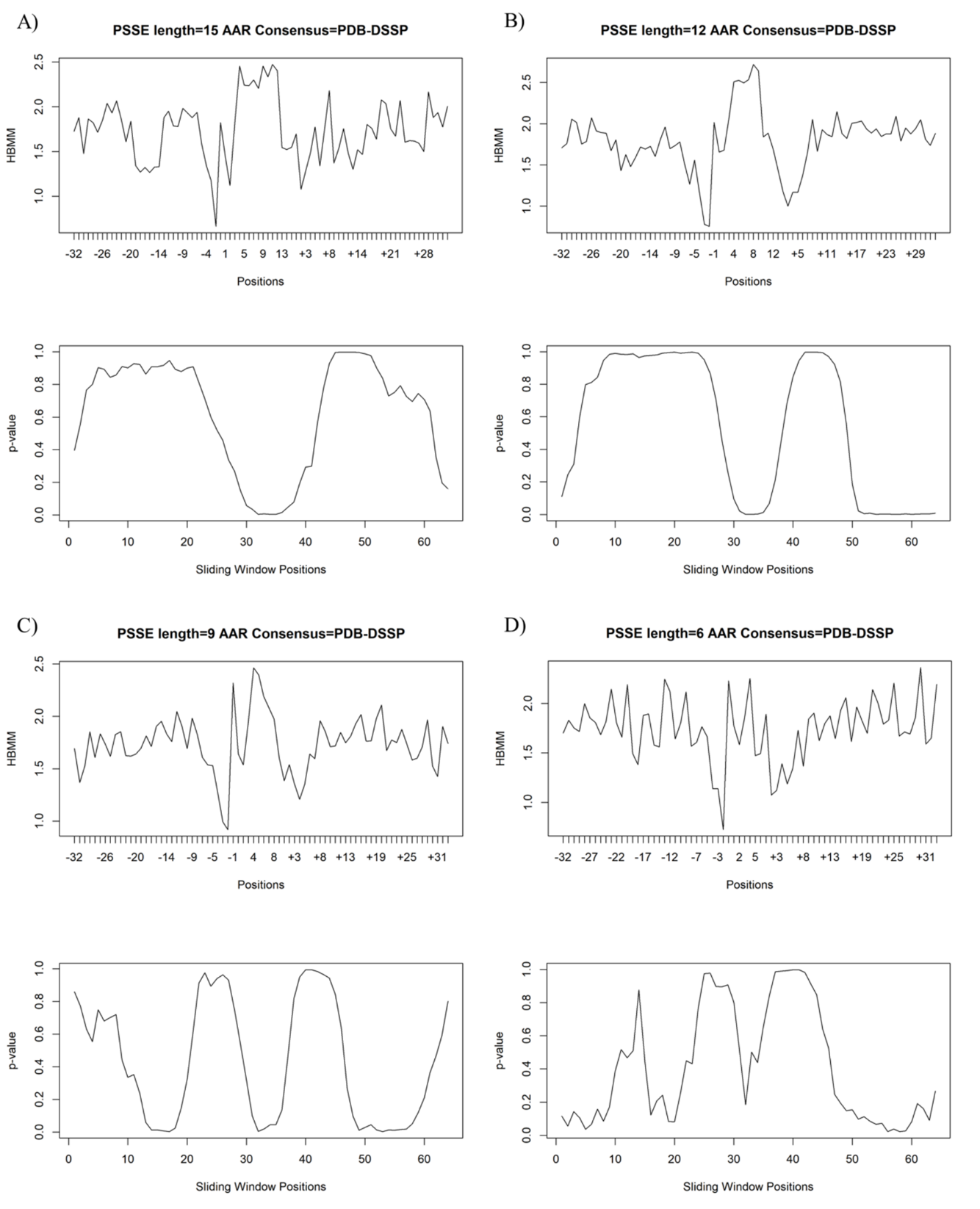


Figure D. HBMM in all-α proteins. Particularly for α-helices with length = 15, 12, 9 and 6 (letters A-D), the region with the PSSE aligned has a specific behaviour, and the establishment of more contacts in this region than outside the PSSE is observed. Immediately before and after the alignment, the plots show a decrease in parameter values. The p-value plots have values near 0 in the PSSE region (A and B). The graphs presented in C and D show that there are zero p-value regions other than the PSSE; these regions are a consequence of the insufficient discerning power of a single descriptor as well as the influence of helices in the flanking regions.


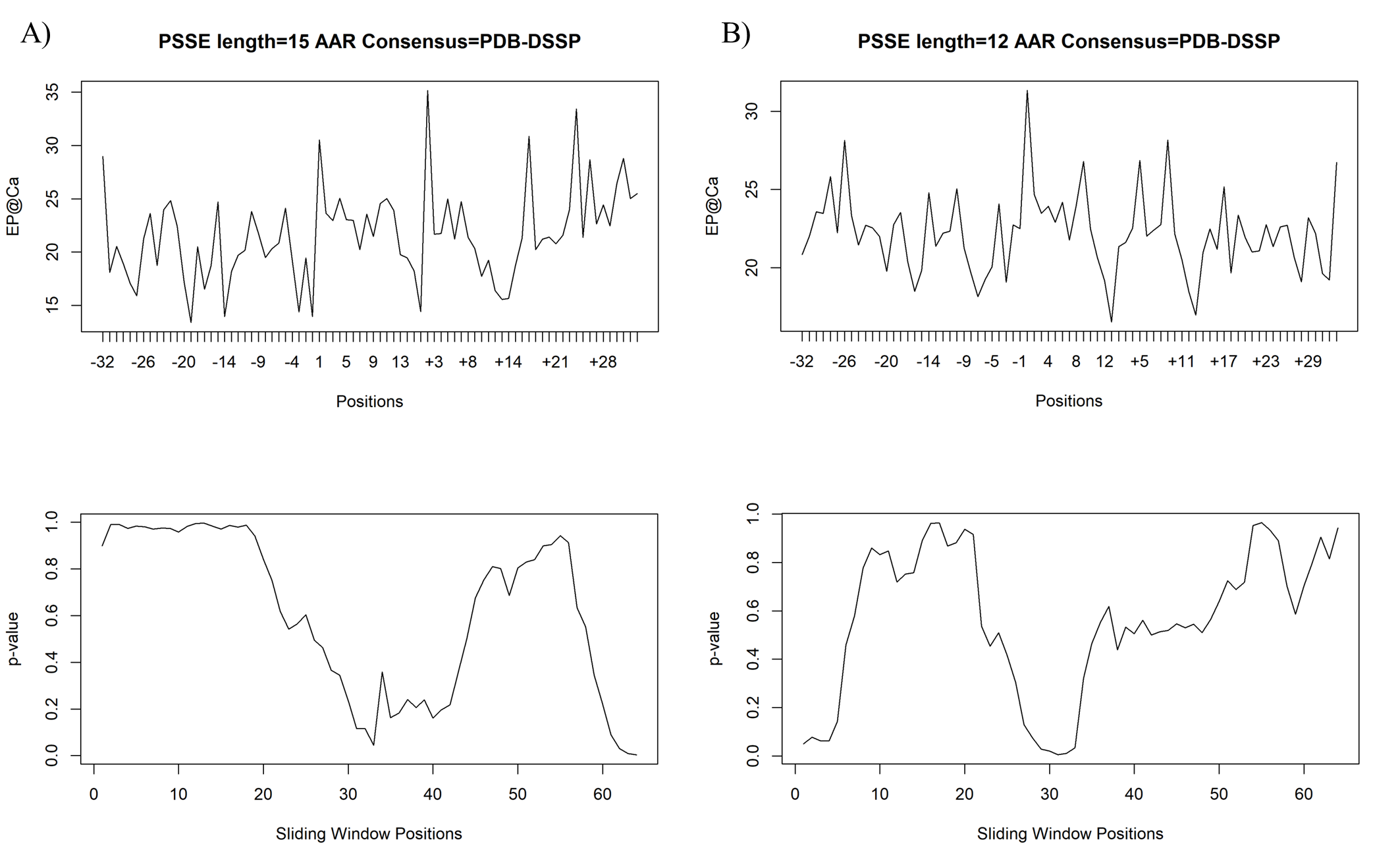


Figure E. EP@Cα in all-α proteins. Particularly for α-helices with length = 15 and 12 (letters A-B), the region with the PSSE aligned has a specific behaviour, and the establishment of more contacts in this region than outside the PSSE is observed. Immediately before and after the alignment, the plot shows that the parameter values decreased. The p-value plots have values near 0 in the PSSE region (letters A and B).

## STING_RDB Descriptors

### Electrostatic Potential

Amino acid residues are made of atoms, and these atoms may have a positive charge. Blue Star STING calculates the electrostatic potential using Delphi software [1], which uses the Poisson-Boltzmann equation:

$$\nabla\cdot\left[ \varepsilon_{r}\left( r \right)\nabla\varphi\left( r \right) \right]=\frac{1}{\varepsilon_{0}}[\rho^{cargasfixas}\left( r \right)-\frac{\varepsilon_{solv}k^{2}\left( r \right)}{4\pi}\varphi(r)]$$

where $\epsilon_{r}\left( r \right)$ is the relative local dielectric constant; $\epsilon_{solv}$ is the solvent dielectric constant; $\rho^{cargas\_fixas}\left( r \right)$ is the distribution of the charge above the solute; and $k^{2}$ is the Debye parameter, which is equal to:

$$k^{2}=\frac{8\pi e^{2}C}{\varepsilon_{solv}k_{B}T}[{kT}/e]$$

where $C$ is the salt mass concentration; $k_{B}$ is the Boltzmann constant; and $T$ is the absolute temperature.

STING_RDB stores the electrostatic potential values calculated for Cα, the last heavy atom (LHA), the average of all atoms of the amino acid residue and the surfaces created for each amino acid residue that is part of the protein surfaces.

### Accessibility

Blue Star STING calculates the accessibility using SurfV software [2]. Three values are calculated: accessibility of the isolated chain, accessibility of the complex with another chain, and relative accessibility. The last value is computed using the values in Table A [3].

| Residue | ASA_1 | ASA_2 | ASA_3 | ASA_4 | Difference = (max-min) | %difference | \|%diff\| | Mult.fact. | BLUE STAR STING ASA VALUE USED = MAX EXP. VALUE X MULT.FACT. |
| --- | --- | --- | --- | --- | --- | --- | --- | --- | --- |
| ALA | 231.680 | 232.768 | 233.585 | 234.345 | 2.665 | 1.14 | 2 | 1.02 | 239.0 |
| ARG | 362.479 | 366.063 | 371.165 | 372.958 | 10.479 | 2.81 | 3 | 1.03 | 384.1 |
| ASN | 277.159 | 278.898 | 282.891 | 284.687 | 7.528 | 2.64 | 3 | 1.03 | 293.2 |
| ASP | 271.343 | 273.849 | 276.055 | 276.055 | 5.484 | 1.98 | 2 | 1.02 | 282.4 |
| CYS | 255.831 | 256.499 | 260.818 | 262.350 | 6.519 | 2.48 | 3 | 1.03 | 270.2 |
| GLN | 302.331 | 303.265 | 309.626 | 310.915 | 8.584 | 2.76 | 3 | 1.03 | 320.2 |
| GLU | 292.397 | 303.215 | 304.521 | 312.052 | 19.655 | 6.30 | 7 | 1.07 | 333.9 |
| GLY | 202.875 | 205.294 | 205.443 | 205.471 | 2.596 | 1.26 | 2 | 1.02 | 209.6 |
| HIS | 303.173 | 305.122 | 307.905 | 317.645 | 14.472 | 4.56 | 5 | 1.05 | 333.5 |
| ILE | 302.749 | 302.944 | 305.736 | 306.417 | 3.668 | 1.20 | 2 | 1.02 | 312.5 |
| LEU | 306.523 | 310.613 | 311.242 | 311.438 | 4.915 | 1.58 | 2 | 1.02 | 317.7 |
| LYS | 339.108 | 340.594 | 343.988 | 347.742 | 8.634 | 2.48 | 3 | 1.03 | 358.2 |
| MET | 317.302 | 317.461 | 321.639 | 325.357 | 8.055 | 2.48 | 3 | 1.03 | 335.1 |
| PHE | 325.050 | 327.259 | 340.984 | 342.137 | 17.087 | 4.99 | 5 | 1.05 | 359.2 |
| PRO | 265.778 | 265.784 | 266.094 | 266.453 | 675 | 0.25 | 1 | 1.01 | 269.1 |
| SER | 241.856 | 242.445 | 243.840 | 245.213 | 3.357 | 1.37 | 2 | 1.02 | 250.1 |
| THR | 265.434 | 266.091 | 267.756 | 269.504 | 4.070 | 1.51 | 2 | 1.02 | 274.9 |
| TRP | 363.036 | 368.853 | 385.297 | 387.278 | 24.242 | 6.26 | 7 | 1.07 | 414.4 |
| TYR | 352.526 | 355.254 | 359.056 | 360.794 | 8.268 | 2.29 | 3 | 1.03 | 371.6 |
| VAL | 276.272 | 278.396 | 279.453 | 280.703 | 4.431 | 1.58 | 2 | 1.02 | 286.3 |

Table A. Accessible surface area calculated using Blue Star STING (<http://www.cbi.cnptia.embrapa.br/SMS/STINGm/help/solvent_accessible_area.html>)

Four PDB files were selected with one of the 20 amino acids present in the C-terminal region of each of these files. Afterward, the accessible area of the surface of this single amino acid was calculated. This value is called the accessible surface area (ASA).

For each of these four PDB files, the maximum and minimum ASA values were identified. The difference between these values was added to the maximum value. These four values are shown in Table A (ASA_1, ASA_2, ASA_3, ASA_4). The values in the blue column are used by Blue Star STING as the ASA values for each amino acid inside the chain.

The accessible area for each amino acid residue was calculated using a probe sphere with radius = 1,4 Å (water molecule radius). This probe sphere corresponds to the protein Van der Waals surface. The surface made by the geometric centre of the probe sphere is the solvent accessible surface (Figure F) [4].


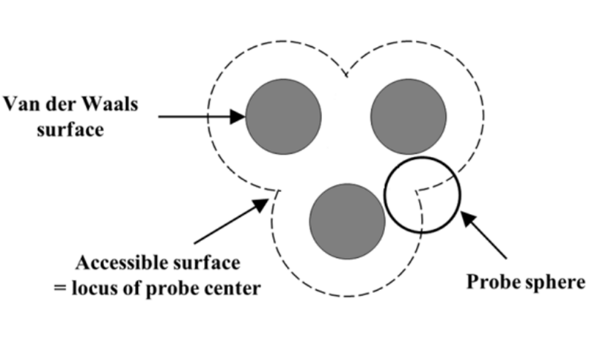


Figure F. Accessible surface according to Van der Waals surface [4].

### Hydrophobicity

An amino acid is hydrophobic when it does not have oxygen and nitrogen atoms in its side chain, and due to this characteristic, the amino acid does not form hydrogen bonds with water molecules. On the other hand, amino acids capable of forming these bonds are called hydrophilic. Hydrophobicity is the measure of how hydrophobic or hydrophilic an amino acid is. This property is fundamental to the stability of the protein. Blue Star STING calculates the hydrophobicity using the Kyte-Doolittle [5] and Radzicka [6] scales. See the equations below below.

$${Hydrophobicity}_{i}=\frac{{Access}_{i}}{{AccessMax}_{i}}\times{kyteDooLittle}_{i}$$

$${Hydrophobicity}_{i}=\frac{{Access}_{i}}{{AccessMax}_{i}}\times{Radzicka}_{i}$$

where ${KyteDooLittle}_{i}$ is the Kyte-Doolittle value of hydrophobicity for each amino acid residue (Table B); ${Radzicka}_{i}$ is the Radzicka value of hydrophobicity for each amino acid residue (Table C); ${Access}_{i}$ is the value of accessibility for amino acid residue$i$; and ${AccessMax}_{i}$ is the maximum accessibility value for amino acid residue $i$ (Table A).

| Amino acid | Side chain polarity | Side chain charge at pH 7,4 | Hydrophobicity constant Kyte-Doolittle |
| --- | --- | --- | --- |
| (Arg) | Polar | Positive | -4.5 |
| (Lys) | Polar | Positive | -3.9 |
| (Asp) | Polar | Negative | -3.5 |
| (Glu) | Polar | Negative | -3.5 |
| (Asn) | Polar | Neutral | -3.5 |
| (Gln) | Polar | Neutral | -3.5 |
| (His) | Polar | Neutral/Positive | -3.2 |
| (Pro) | Nonpolar | Neutral | -1.6 |
| (Tyr) | Polar | Neutral | -1.3 |
| (Trp) | Nonpolar | Neutral | -0.9 |
| (Ser) | Polar | Neutral | -0.8 |
| (Thr) | Polar | Neutral | -0.7 |
| (Gly) | Nonpolar | Neutral | -0.4 |
| (Ala) | Nonpolar | Neutral | 1.8 |
| (Met) | Nonpolar | Neutral | 1.9 |
| (Cys) | Nonpolar | Neutral | 2.5 |
| (Phe) | Nonpolar | Neutral | 2.8 |
| (Leu) | Nonpolar | Neutral | 3.8 |
| (Val) | Nonpolar | Neutral | 4.2 |
| (Ile) | Nonpolar | Neutral | 4.5 |

Table B. Kyte-Doolittle hydrophobicity scale [5].

| Amino acid | Side chain polarity | Side chain charge at pH 7,4 | Hydrophobicity constant Radzicka |
| --- | --- | --- | --- |
| (Arg) | Polar | Positive | -14.92 |
| (Asp) | Polar | Negative | -8.72 |
| (Glu) | Polar | Negative | -6.81 |
| (Asn) | Polar | Neutral | -6.64 |
| (Lys) | Polar | Positive | -5.55 |
| (Gln) | Polar | Neutral | -5.54 |
| (His) | Polar | Neutral/Positive | -4.66 |
| (Ser) | Polar | Neutral | -3.4 |
| (Thr) | Polar | Neutral | -2.57 |
| (Tyr) | Polar | Neutral | -0.14 |
| (Gly) | Nonpolar | Neutral | 0.94 |
| (Cys) | Nonpolar | Neutral | 1.28 |
| (Ala) | Nonpolar | Neutral | 1.81 |
| (Trp) | Nonpolar | Neutral | 2.33 |
| (Met) | Nonpolar | Neutral | 2.35 |
| (Phe) | Nonpolar | Neutral | 2.98 |
| (Pro) | Nonpolar | Neutral | 3.5 |
| (Val) | Nonpolar | Neutral | 4.04 |
| (Ile) | Nonpolar | Neutral | 4.92 |
| (Leu) | Nonpolar | Neutral | 4.92 |

Table C. Radzicka hydrophobicity scale [6].

### Contacts

Interactions between the amino acids of the protein are indispensable to maintain its stability. Blue Star STING defines the contacts between the amino acid residues based on their relative distances. The method is as follows: a) classify the atoms in groups according to their electrostatic behaviour and position in the main or side chain; b) select the atoms based on the type of contact the atom may make and use the experimentally defined distance restriction for each type of contact. Table D presents the five types of contacts stored in STING_RDB and their energies [7].

| Contact type | Contact energy (kcal mol^-1^) |
| --- | --- |
| Hydrophobic interactions | 0.6 |
| Aromatic stacking | 1.5 |
| Hydrogen bonds ^(*)^ | 2.6 |
| Salt bridge ^(†)^ | 10.0 |
| SS bond | 85.0 |

Table D. Contact types and their energies (*). There are nine hydrogen bonds: main chain–main chain, main chain–(1 H_2_O)–main chain, main chain–(2 H_2_O)–main chain, main chain–side chain, main chain–(1 H_2_O)–side chain, main chain–(2 H_2_O)–side chain, side chain–side chain, side chain–(1 H_2_O)–side chain, and side chain–(2 H_2_O)–side chain. (†) Salt bridge may be attractive or repulsive [7].

### Cross Link Order

Amino acid residues separated by a large distance in the primary structure (amino acid sequence) may make contact with one another in the 3D structure. Blue Star STING calls this contact a “cross link.” The procedure for calculating cross links is as follows: amino acid residues that are far from each other (separated by 15, 20 or 30 positions) in the sequence but are inside a probe sphere within a radius of 3,5 Å, 5 Å and 8,5 Å in the 3D structure may make contact with one another under the conditions explained in the previous section (Contacts). The order of the cross links is the number of contacts made by the same amino acid residue (Panel A in Figure G).

### Cross-presence order

Similar to the cross link order, the cross presence order sums all amino acid residues inside the probe sphere with a radius of 3,5 Å, 5 Å and 8,5 Å, even if these residues do not make contact. The order is the number of amino acid residues inside the probe sphere (Panel B in Figure G).


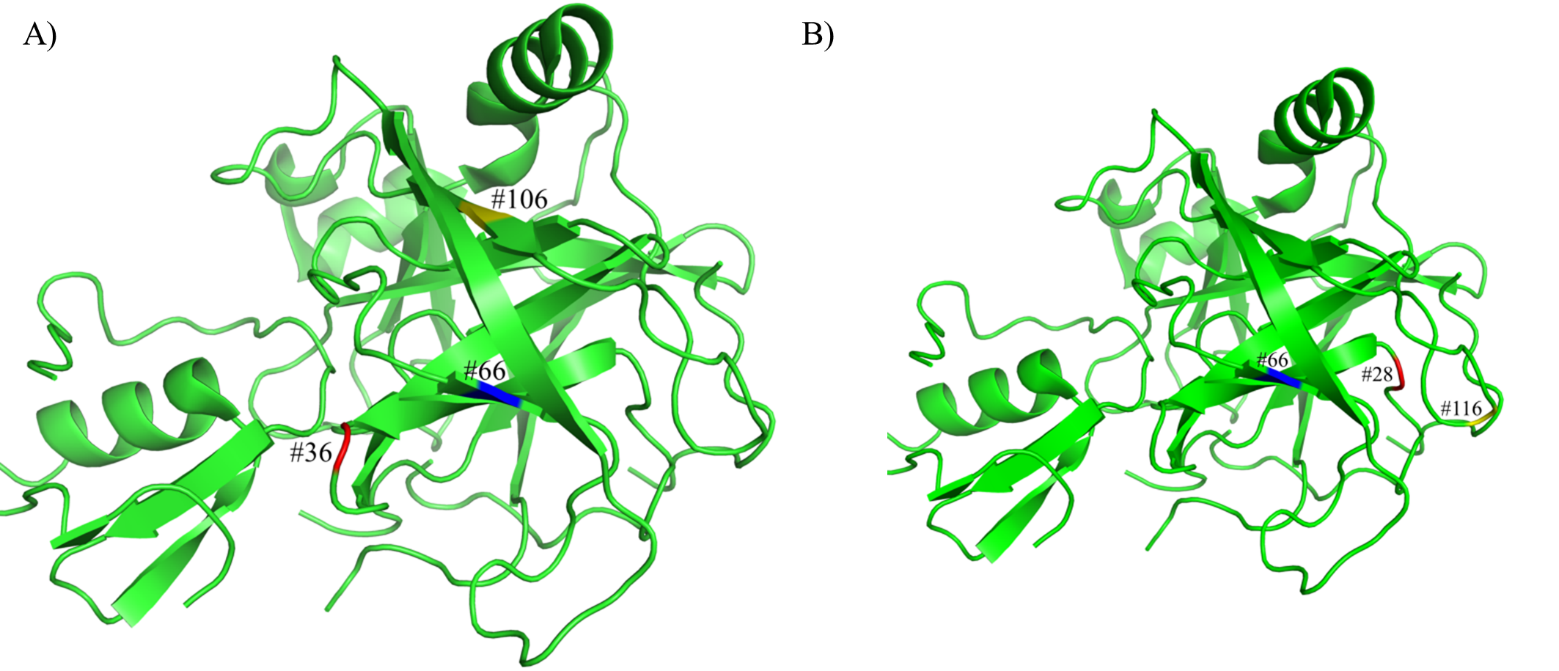


Figure G (A) Cross link. PDB: 1CHO CHAIN: F. Amino acid residue 36 (red) contacts residues 66 (blue) and 106 (yellow). Although these residues are far from one another in the sequence, the residues are inside the 3,5 Å probe sphere and make contact with each other. (B) Cross presence. PDB: 1CHO CHAIN: F. Although these residues do make contact, amino acid residues 28 (red), 66 (blue) and 116 (yellow) are inside the probe sphere, even though distant in the sequence. Pictures were made using PyMOL.

### Dihedral angles

Blue Star STING calculates the dihedral angles φ and ψ using the Ramachandran method [8]. See the equation below.

$$\varphi={tg}^{-1}[2\left( v,u \right)]\times57,29578$$

where arctan $2\left( v,u \right)$is a function that returns the arc-tangent of *v*/*u* in radians; *v* and *u* are auto vectors with the minor relative auto value; and 57.29578 is the constant used to convert radians into degrees.

### Rotamers

As explained in the previous section (Dihedral Angles), amino acid residue atoms on the main chain feature dihedral angles φ and ψ. The atoms present on the side chain may have up to five angles (χ-1, χ-2, χ-3, χ-4, χ-5) depending on the length of the side chain. However, since the side chain has different lengths, not all amino acid residues have the same number of angles, χ. For example, χ-5 is calculated just for arginine residues, which have six atoms in the side chain.

Because the dihedral angles φ and ψ have a more preferred statistical configuration, the side chain angles have values which are statistically more preferred [9].

### Unused contacts

Blue Star STING calculates and stores in STING_RDB the number of contacts made between the amino acid residues inside a protein (see the section Contacts). It is possible to define the maximum number of contacts for each type of amino acid for each of the fourteen types of contacts (Table D). The descriptor “unused contacts” is calculated by for each amino acid residue as the number of contacts minus the maximum number of contacts found in STING_RDB for each type of contact. This descriptor describes possible unused contacts and defines the type of contacts that could be made but were not.

### Temperature factor

This descriptor is extracted directly from the PDB file. This value indicates the mobility level of the atoms inside the crystal. A temperature factor of less than 30 Å^2^ means that the atoms do not move too much, and the atoms are in the same position as for the other molecules inside the crystal. Values larger than 60 Å^2^ imply that the atom moves so much that the atom cannot be viewed using X-ray crystallography [10]. This situation frequently happens for the atoms on the surface of the protein where the long side chains on the loops are freer to oscillate in space.

### Weighted Neighbour Averages

Weighted neighbour average (WNA) is the name given to the descriptor *D* weighted by the values for the neighbouring residues. The results are associated with the amino acid residue of interest using the equations below.

$$D_{WNA}^{Surf}=\sum_{i=0}^{N} D_{i}{Acc}_{relative}$$

$$D_{WNA}^{Dist}=D_{0}+\sum_{i=1}^{N} \frac{D_{i}}{d_{i}}$$

where *D_0_* is the value of descriptor *D* for the amino acid residue of interest, while the other *D_i_* are the values of the same descriptor for the *N* spatial neighbours;${Acc}_{relative}$ is the relative accessible area for the solvent; and *d_i_* is the distance from the *i^th^* neighbour to the amino acid residue of interest. S6 Equation means that amino acid residues with a higher percentage of accessible area have more influence on the amino acid residue of interest, while S7 Equation means that the larger the spatial distance between amino acid residues is, the less influence these residues have on the amino acid residue of interest, regardless of the accessibility of each amino acid.

The algorithm used to calculate the WNA values was defined by Porollo and Meller [11]. The algorithm specifies a probe sphere with a radius of 15 Å, centred on each amino acid Cα for each protein structure stored in STING_RDB. According to Silveira [12], beyond the 15 Å distance, the neighbour's influence drops to zero.

## Sliding Window Test: variations in window size


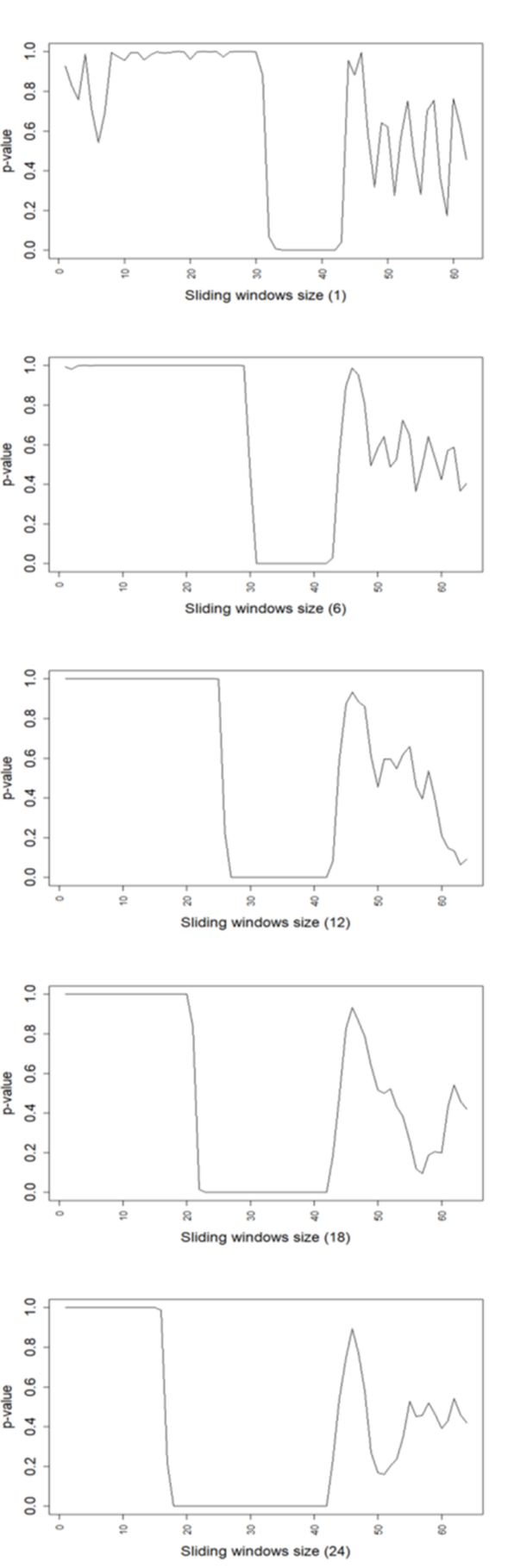


Figure H. Tests with different sizes of sliding windows (1, 6, 12, 18 and 24) for a helix of 12 AARs demonstrated that the sliding window with the minimum size (1) hits exactly at the analysed helix position (region where the p-value is approximately equal to zero).

## References

1. Rocchia W, Sridharan S, Nicholls A, Alexov E, Chiabrera A, Honig B. Rapid grid-based construction of the molecular surface for both molecules and geometric objects: applications to the finite difference Poisson-Boltzmann method. Journal of Computational Chemistry. 2002: p. 128-137.

2. Sridharan S, Nicholls A, Honig B. A new vertex algorithm to calculate solvent accessible surface-areas. Faseb Journal. 1992: p. A174-A174.

3. Goran N, Higa HR, Luiz MA, Kuser FP. Calculo de Área acessivel por Solvente Utilizando SURFV - Definição de Interface Intramolecular pelo SMS. Comunicado Técnico. Campinas SP: Embrapa Informática Agropecuária; 2002. Report No.: 36.

4. Lee B, Richards FM. The interpretation of protein structures: estimation of static accessibility. Journal of molecular biology. 1971: p. 379-IN4.

5. Kyte J, Doolittle. RF. A simple method for displaying the hydropathic character of a protein. Journal of molecular biology. 1982: p. 105-132.

6. Radzicka A, Wolfenden R. Comparing the polarities of the amino acids: side-chain distribution coefficients between the vapor phase, cyclohexane, 1-octanol, and neutral aqueous solution. Biochemistry. 1988: p. 1664-1670.

7. Mancini AL, Higa RH, Oliveira A, Dominiquini F, Kuser PR, Yamagishi MEB, et al. STING Contacts: a web-based application for identification and analysis of amino acid contacts within protein structure and across protein interfaces. Bioinformatics. 2004: p. 2145-2147.

8. Ramachandran GN, Ramakrishnan C, Sasisekharan V. Stereochemistry of polypeptide chain configurations. Journal of molecular biology. 1963: p. 95-99.

9. Schrauber H, Eisenhaber F, Argos P. Rotamers: to be or not to be?: an analysis of amino acid side-chain conformations in globular proteins. Journal of molecular biology. 1993: p. 592-612.

10. from:l. PP[[2j1A. Proxychem. [Online]. [cited 2018 June 7. Available from: http://www.proxychem.com/macromolecular_crystallography.htm.

11. Porollo A, Meller J. Prediction‐based fingerprints of protein–protein interactions. Proteins: Structure, Function, and Bioinformatics. 2007: p. 630-645.

12. Silveira CHd, Pires DEV, Minardi RC, Ribeiro C, Veloso CJM, Lopes JCD, et al. Protein cutoff scanning: A comparative analysis of cutoff dependent and cutoff free methods for prospecting contacts in proteins.. Proteins: Structure, Function, and Bioinformatics. 2009: p. 727-743.
